# Activated FGF2 signaling pathway in tumor vasculature is essential for acquired resistance to anti-VEGF therapy

**DOI:** 10.1038/s41598-020-59853-z

**Published:** 2020-02-19

**Authors:** Kenji Ichikawa, Saori Watanabe Miyano, Yukinori Minoshima, Junji Matsui, Yasuhiro Funahashi

**Affiliations:** Tsukuba Research Laboratories, Eisai Co., Ltd., Tsukuba, Ibaraki, 5-1-3 Tokodai, Tsukuba, Ibaraki, 300-2635 Japan

**Keywords:** Cancer microenvironment, Tumour angiogenesis, Cancer therapeutic resistance

## Abstract

Anti–vascular endothelial growth factor (VEGF) therapy shows antitumor activity against various types of solid cancers. Several resistance mechanisms against anti-VEGF therapy have been elucidated; however, little is known about the mechanisms by which the acquired resistance arises. Here, we developed new anti-VEGF therapy–resistant models driven by chronic expression of the mouse VEGFR2 extracellular domain fused with the human IgG4 fragment crystallizable (Fc) region (VEGFR2-Fc). In the VEGFR2-Fc–expressing resistant tumors, we demonstrated that the FGFR2 signaling pathway was activated, and pericytes expressing high levels of FGF2 were co-localized with endothelial cells. Lenvatinib, a multiple tyrosine kinase inhibitor including VEGFR and FGFR inhibition, showed marked antitumor activity against VEGFR2-Fc–expressing resistant tumors accompanied with a decrease in the area of tumor vessels and suppression of phospho-FGFR2 in tumors. Our findings reveal the key role that intercellular FGF2 signaling between pericytes and endothelial cells plays in maintaining the tumor vasculature in anti-VEGF therapy–resistant tumors.

## Introduction

Anti-vascular endothelial growth factor (VEGF) therapy, such as anti-VEGF antibody (Ab), anti-VEGFR2 Ab, and multiple tyrosine kinase inhibitors (TKIs) targeting VEGFR2, has been used to treat various types of solid cancers for over a decade. Despite massive efforts, the mechanisms of acquired resistance to anti-VEGF therapy are still not completely understood^1,2^.

Other angiogenic factors such as fibroblast growth factors (FGFs)^3,4^, angiopoietins^5,6^, and platelet-derived growth factors (PDGFs)^7–9^ have been reported to induce tumor angiogenesis as a single factor or in crosstalk with VEGF^10^. Upregulation of FGFs by chronic anti-VEGF therapy has been reported in the RIP1-Tag2 mouse (a pancreatic neuroendocrine tumor model) treated by DC101 (an anti-VEGFR2 Ab)^11^, and in a human head and neck squamous cell carcinoma xenograft tumor model treated by bevacizumab (an anti-VEGFA Ab)^12^. In a study of MCaIV syngeneic tumor models, cancer-associated fibroblasts and adipocytes expressed FGF2 and mediated resistance to anti-mouse VEGF Ab B20^13^. In addition, in the Y-MESO-14 (human malignant pleural mesothelioma cell line) xenograft tumor model, fibrocyte-like cells mediated the resistance to bevacizumab by producing FGF2^14^. In contrast, concurrent inhibition of VEGFR and FGFR by a chimeric dual decoy receptor enhanced antitumor activity in A549 (human lung cancer cell line) and Caki-1 (human renal cancer cell line) xenograft tumor models^15^. These results suggest that the FGF signaling pathway contributes to resistance to anti-VEGF therapy; however, it is largely unknown how the FGF signaling pathway becomes activated when tumors acquire resistance to anti-VEGF therapy. Recently, the role of the VEGF signaling pathway in cancer immunity has received increased attention because of the promising combination antitumor activity of anti-VEGF therapy with immune checkpoint inhibitors in pre-clinical models^16,17^ and clinical trials^18,19^. Therefore, it has become more important to understand the mechanism for acquired resistance to anti-VEGF therapy.

Here, we developed novel tumor models for acquired resistance to anti-VEGF therapy by expressing a fusion protein of the mouse VEGFR2 extracellular domain and human IgG4 fragment crystallizable (Fc) region (VEGFR2-Fc) in Renca mouse renal cell carcinoma and B16F10 mouse melanoma cell lines. Using these acquired-resistance models, we evaluated tumor angiogenesis and activated cell signaling pathways by immunohistochemistry analysis and differentially expressed gene analysis, respectively. From the results, we identified activation of the FGF signaling pathway in tumor vasculature as playing a major role in resistance to anti-VEGF therapy. In addition, dual inhibition of FGFR and VEGFR overcame resistance to anti-VEGF therapy and enhanced antitumor activity as well as antiangiogenesis activity in these models.

## Results

### Establishment of anti-VEGF therapy–resistant model by overexpressing VEGFR2-Fc

To establish anti-VEGF therapy–resistant models, we introduced Mock vector or an expression vector for the VEGFR2-Fc fusion gene (Supplementary Fig. 1a) into Renca and B16F10 cells. Supernatants from cell cultures of established tumor cells were collected to assess the specific binding of VEGFR2-Fc (Supplementary Fig. 1b) to VEGF. VEGFR2-Fc interacted with VEGF, but not with FGF1 or FGF2 (Supplementary Fig. 1c,d). To examine the biological functions of VEGFR2-Fc, we performed a VEGF-induced sandwich tube formation assay with human umbilical vein endothelial cells (HUVECs) by co-culturing with Mock or VEGFR2-Fc–expressing tumor cells (Supplementary Fig. 1e). The tumor cells were plated on top of two layers of collagen gels, between which HUVECs were embedded. VEGF-induced tube formation was significantly abrogated by co-culture with VEGFR2-Fc–expressing tumor cells (Supplementary Fig. 1 f–i) in both Renca and B16F10 models.

**Figure 1:**
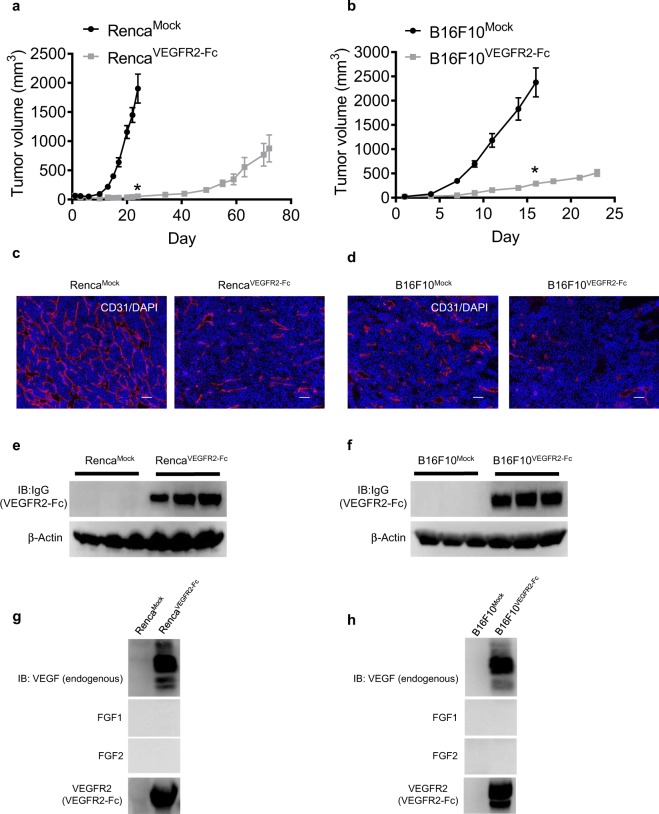
*In vivo* tumor growth and angiogenesis of Mock and VEGFR2-Fc–expressing tumors. (**a**,**b**) *In vivo* tumor growth of Mock and VEGFR2-Fc–expressing tumors. (**a**) Renca model; (**b**) B16F10 model. Data are means ± SEM (n = 7 in Renca model and n = 8 in B16F10 model). *p < 0.05 (unpaired *t*-test). Comparison of tumor volumes between Mock and VEGFR2-Fc–expressing groups was conducted for the last time point of the Mock group in (**a**) Day 24 and (**b**) Day 16. (**c**,**d**) Tumor angiogenesis of Mock and VEGFR2-Fc–expressing tumors. Representative images of CD31 staining (marker of endothelial cells; red) and DAPI (4′,6-diamidino-2-phenylindole) (marker of DNA; blue). (**c**) Renca model; (**d**) B16F10 model. Scale bars represent 100 μm. (**e**,**f**) Expression of VEGFR2-Fc (detected by anti-IgG Ab) in tumors resected at volumes of ~500 mm^3^. (**e**) Renca model; (**f**) B16F10 model. Three independent tumors were analyzed. (**g,h**) Immunoprecipitation assay for co-precipitation of VEGFR2-Fc with endogenous VEGF, FGF1, or FGF2 in Mock or VEGFR2-Fc–expressing tumor lysates. (**g**) Renca model; (**h**) B16F10 model. IB, Immunoblotting.

To evaluate *in vivo* tumor growth, we subcutaneously inoculated Mock and VEGFR2-Fc–expressing tumor cells into syngeneic mice. Renca^VEGFR2-Fc^ and B16F10^VEGFR2-Fc^ tumor growths *in vivo* were significantly delayed compared with those of Renca^Mock^ and B16F10^Mock^ tumors, respectively (Fig. 1a,b). In contrast, the *in vitro* 2D cell growth curves of Mock and VEGFR2-Fc–expressing tumor cells were almost identical to each other (Supplementary Fig. 1j,k). Next, we evaluated tumor angiogenesis in Renca^VEGFR2-Fc^ and B16F10^VEGFR2-Fc^ tumors by staining endothelial cells with anti-CD31 Ab. VEGFR2-Fc–expressing tumors displayed suppressed tumor angiogenesis with a decrease in the area of tumor vessels compared with Mock tumors (Fig. 1c,d); however, some angiogenesis still occurred. By conducting Western blot analysis of tumor lysates from tumors resected at a volume of ~500 mm^3^, we confirmed that expression of VEGFR2-Fc was maintained in Renca^VEGFR2-Fc^ and B16F10^VEGFR2-Fc^ tumors (Fig. 1e,f). In immunoprecipitation analysis, VEGFR2-Fc in the tumors specifically interacted with endogenous VEGF, but not with FGF1 or FGF2 (Fig. 1g,h). To exclude the possibility that soluble Fc protein effects tumor growth or angiogenesis, transfectants stably expressing soluble Fc were established (Supplementary Fig. 2a,b). Mock and soluble Fc–expressing tumor cells had similar *in vivo* tumor growth (Supplementary Fig. 2c,d) and tumor angiogenesis (Supplementary Fig. 2e,f) in both Renca and B16F10 models, indicating that IgG4 Fc protein does not harbor antitumor or antiangiogenic activity. These results suggest that Renca^VEGFR2-Fc^ and B16F10^VEGFR2-Fc^ tumors keep growing *in vivo* by escaping from abrogation of the VEGF signaling pathway, even though tumor growth was delayed due to inhibition of tumor angiogenesis by VEGFR2-Fc. Therefore, we defined the VEGFR2-Fc–expressing Renca and B16F10 tumors as anti-VEGF resistant models.

### Identification of activated cell signaling pathways in VEGFR2-Fc–expressing tumors

**Figure 2:**
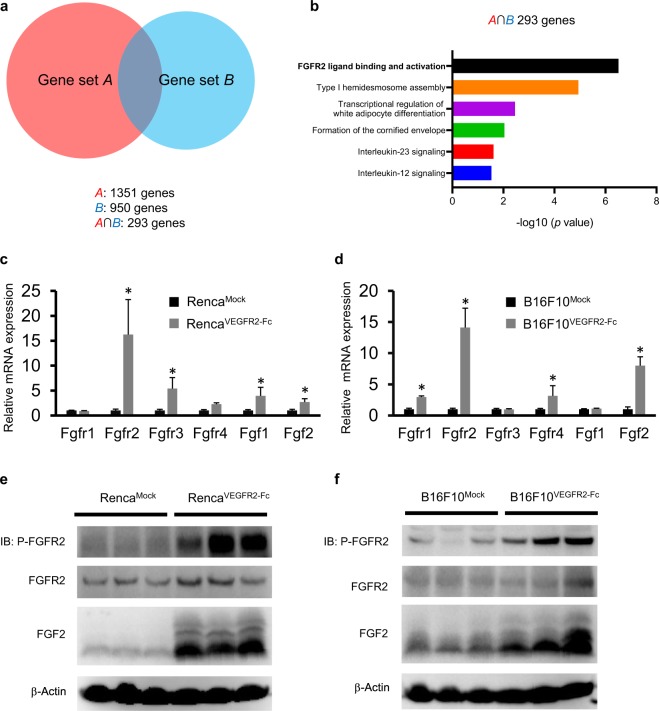
Pathway analysis in VEGFR2-Fc–expressing tumors compared with Mock tumors based on RNA-Seq analysis. (**a**) Venn diagram showing genes that are more than 1.5 times upregulated in VEGFR2-Fc–expressing tumors compared with Mock tumors. Gene set *A* is defined as upregulated in Renca^VEGFR2-Fc^ compared with Renca^Mock^. Gene set *B* is defined as upregulated in B16F10^VEGFR2-Fc^ compared with B16F10^Mock^. *A* ∩ *B* shows area of intersection of gene set *A* and gene set *B*. (**b**) Reactome analysis of signaling pathways in *A* ∩ *B*. The top 6 pathways are shown. **(c**,**d**) RT–qPCR analysis of the mRNA levels of various FGF and FGFR family members in Mock and VEGFR2-Fc–expressing tumors. (**c**) Renca model; **(d**) B16F10 model. Data are means ± SEM. *p < 0.05 (unpaired *t*-test). (**e**,**f**) Levels of phospho-FGFR2, FGFR2, FGF2 in lysates extracted from Mock and VEGFR2-Fc tumors analyzed by Western Blot analysis. (**e**) Renca mod**e**l; (**f**) B16F10 model.

To identify activated cell signaling pathways in tumors after chronic exposure to VEGFR2-Fc, RNA-Seq analysis was performed by using Mock and VEGFR2-Fc**–**expressing tumors. We identified 1351 genes (gene set *A*) in the Renca model and 950 genes (gene set *B*) in the B16F10 model that were highly expressed in VEGFR2-Fc–expressing tumors compared with Mock tumors (Fig. 2a). The overlap genes between gene set A and B comprised 293 genes (*A* ∩ *B*). Pathway analysis of these 293 genes identified ‘FGFR2 ligand binding and activation’ pathway (with the lowest *p* value) (Fig. 2b). In addition, this pathway ranked as the top signaling pathway in each separate gene set (Supplementary Fig. 3a,b). These results suggest that the FGFR2 signaling pathway was activated in Renca^VEGFR2-Fc^ and B16F10^VEGFR2-Fc^ tumors as a common acquired resistance mechanism against anti-VEGF therapy.

We then examined mRNA levels of FGF ligands and FGF receptors in the RNA-Seq data. Consistent with the result of the pathway analysis, expression levels of Fgf2 and Fgfr2 mRNA were commonly increased in VEGFR2-Fc–expressing tumors compared with Mock tumors in both the Renca and B16F10 models (Supplementary Fig. 3c,d). When we examined other genes related to tumor angiogenesis (Supplementary Fig. 3e,f), we found no pro-angiogenic receptors and ligands that were consistently upregulated in both models. To confirm the increased mRNA levels of FGF ligands and FGF receptors, we conducted real-time quantitative reverse transcription PCR (RT-qPCR) analysis. Consistent with the RNA-Seq analysis, Fgf2 and Fgfr2 mRNA levels were significantly upregulated in VEGFR2-Fc–expressing tumors compared with Mock tumors in both models. The mRNA levels of some of other FGF ligands and FGF receptors were also significantly upregulated in either the Renca or B16F10 model but not both (Fig. 2c,d). To examine the activation status of FGFR2 and the levels of FGF2 and FGFR2 in Mock and VEGFR2-Fc–expressing tumors, we conducted Western blot analysis of tumor lysates from tumors resected at a volume of ~500 mm^3^. FGFR2 was highly phosphorylated, and FGF2 levels were upregulated in VEGFR2-Fc–expressing tumors, compared with Mock tumors in both the Renca and B16F10 models (Fig. 2e,f), although FGFR2 protein levels were not markedly altered. Thus, the FGF2 signaling pathway is a candidate for involvement in acquired resistance to anti-VEGF therapy.

In contrast, Fgf2 mRNA expression level was not significantly different between Mock and VEGFR2-Fc–expressing tumor cells *in vitro*, and Fgfr2 mRNA expression was not detected (Supplementary Fig. 3g,h). This result suggests that the observed increase of Fgf2 and Fgfr2 mRNA in VEGFR2-Fc–expressing tumors *in vivo* (Fig. 2c,d) may be due to stromal cells which include endothelial cells, pericytes, cancer-associated fibroblasts, and tumor infiltrated lymphocytes rather than cancer cells.

### Upregulation of FGF2 in pericytes covering endothelial cells in VEGFR2-Fc–expressing tumors

**Figure 3:**
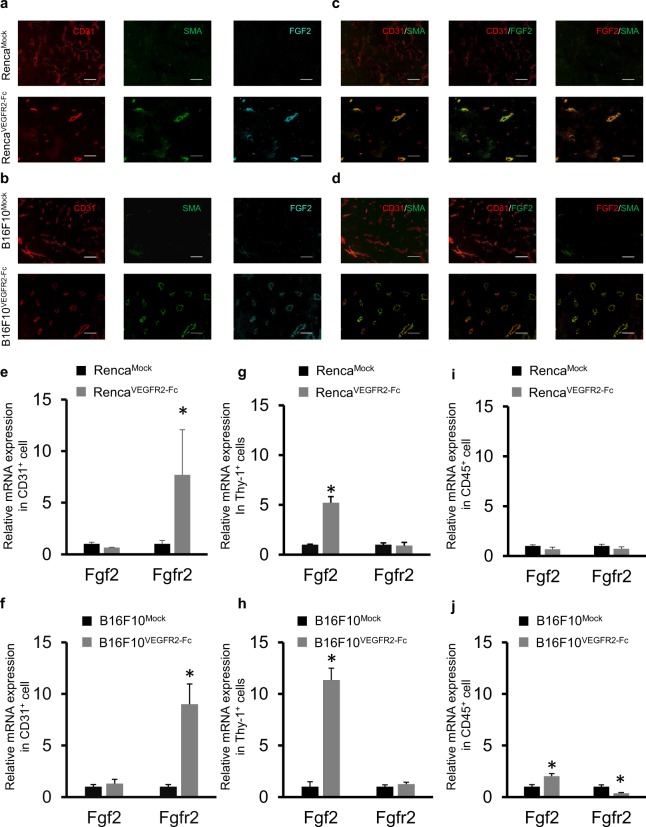
Upregulation of Fgf2 in pericytes covering endothelial cells in VEGFR2-Fc–expressing tumors. (**a**–**d**) Immunofluorescence analysis of CD31 (endothelial cell marker), SMA (pericyte marker), and FGF2. Representative images of Mock and VEGFR2-Fc tumors in Renca and B16F10 models. Tumor sections were stained with anti-CD31 Ab (red), anti-SMA Ab (green), or anti-FGF2 Ab (cyan). As pseudo-colors, green and red were allocated for overlay. Scale bars represent 100 μm. (**e**–**j**) RT-qPCR analysis of Fgf2 and Fgfr2 mRNA in cells isolated from Mock or VEGFR2-Fc–expressing tumors by using microbeads. (**e**,**f**) CD31^**+**^ cells; (**g**,**h**) Thy-1^**+**^ cells; (**i**) and (**j**) CD45^+^ cells. (**e**,**g**,**i**) Renca model; (**f**,**h**,**j**) B16F10 model. Data are means ± SEM (n = 5). *p < 0.05 (unpaired *t*-test).

To investigate the mechanism of activation of the FGFR2 signaling pathway by upregulated FGF2, we conducted immunofluorescence staining using anti-FGF2 Ab, anti-CD31 Ab (for endothelial cells), and anti-SMA Ab (for pericytes) in the Mock and VEGFR2-Fc expressing tumors. CD31 staining was observed in both Mock and VEGFR2-Fc–expressing tumors (Fig. 3a,b). In contrast, SMA staining and FGF2 staining were barely observed in Renca^Mock^ or B16F10^Mock^ tumors, but were clear in Renca^VEGFR2-Fc^ and B16F10^VEGFR2-Fc^ tumors (Fig. 3a,b). The strong FGF2 staining in VEGFR2-Fc–expressing tumors was consistent with the results of Western blot analysis (Fig. 2e,f). Interestingly, SMA-stained cells were adjacent to CD31-stained cells and thus seemed to be aligned to tumor endothelial cells in the VEGFR2-Fc–expressing tumors (Fig. 3c,d). Moreover, FGF2-positive areas were also adjacent to CD31-positive (CD31^**+**^) cells, and SMA-positive (SMA^**+**^) cells might be co-localized with FGF2-positive areas in the VEGFR2-Fc–expressing tumors (Fig. 3c,d). These results suggest that the tumor microenvironment was changed by chronic treatment with VEGFR2-Fc and that anti-VEGF therapy resistant tumor vessels were covered with pericytes, which produce FGF2 to activate the FGFR2 signaling pathway.

To further examine which cell types expressed FGF2 and FGFR2 in tumor microenvironments, we isolated CD31^**+**^ cells (as endothelial cells), Thy-1–positive (Thy-1^**+**^) cells (as pericytes), and CD45-positive (CD45^+^) cells (as tumor-infiltrating lymphocytes) that are major components in tumor stroma cells by using microbeads coated with each respective Ab. Total RNA was then collected from the isolated cells, and mRNA levels of Fgf2, Fgfr2, Pecam1 (CD31), Acta2 (SMA), and Ptprc (CD45) were analyzed by RT-qPCR (Supplementary Fig. 4a–d). Although a small portion of CD45^+^ cells were nonspecfically included by isolation of the anti-CD31 and anti-Thy-1 microbeads, the results confirmed that the microbeads mainly enriched the expected cell population. The isolated Thy-1^+^ cells showed high expression of Acta2 mRNA as expected for pericytes^20^ (Supplementary Fig. 4a–d). Because SMA^+^ cells were mainly adjacent to CD31^+^ cells in VEGFR2-Fc expressing tumors (Fig. 3c,d), we regarded Thy-1^+^ cells as pericytes. The isolated CD31^**+**^cells showed significantly higher Fgfr2 mRNA expression in the VEGFR2-Fc–expressing tumors than in the Mock tumors (Fig. 3e,f), whereas the isolated Thy-1^+^ cells showed significantly higher expression of Fgf2 mRNA in the VEGFR2-Fc–expressing tumors (Fig. 3g,h). For CD45^+^ cells, there was no significant difference in the levels of Fgf2 and Fgfr2 mRNA between Mock and VEGFR2-Fc–expressing tumors in the Renca tumor model (Fig. 3i). In the B16F10 model, for CD45^+^ cells, Fgf2 mRNA levels were significantly upregulated, and Fgfr2 mRNA levels were decreased in the VEGFR2-Fc–expressing tumors compared with in the Mock tumors, although the level changes were small (Fig. 3j). These results show that pericytes were the main cell type that produced FGF2 in the VEGFR2-Fc–expressing tumors. Therefore, FGF2 supplied by pericytes may effectively activate FGFR2 in endothelial cells in a juxtacrine manner, and induce an escape pathway from anti-VEGF therapy in the acquired resistant models.

### Effects of simultaneous expression of VEGFR2-Fc and FGFR2-Fc on *in vivo* tumor growth and tumor angiogenesis in the Renca and B16F10 models

**Figure 4:**
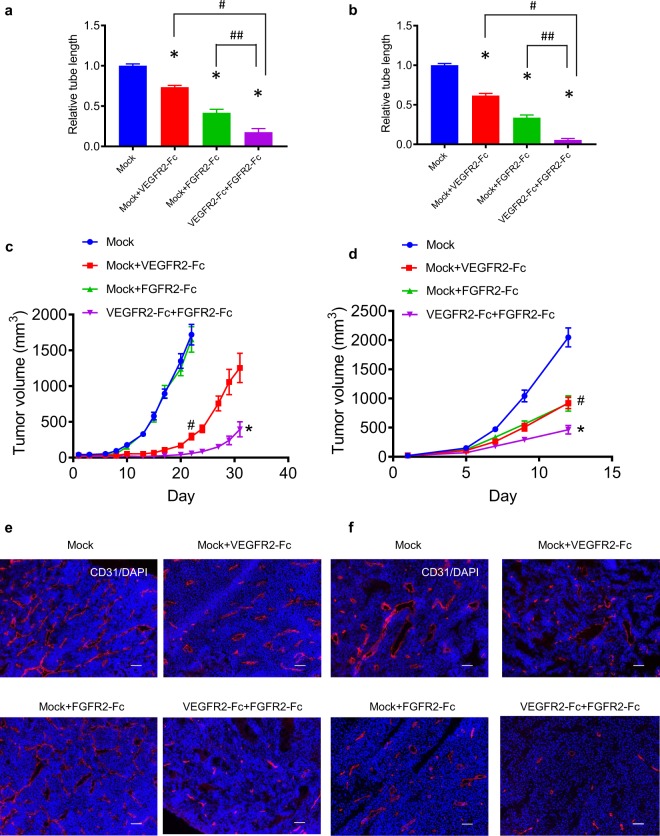
*In vivo* tumor growth and tumor angiogenesis of Mock, VEGFR2-Fc, and FGFR2-Fc–expressing tumors. (**a**,**b**) *In vitro* HUVEC sandwich tube formation by co-culture with a mixture of Mock and either VEGFR2-Fc– or FGFR2-Fc–expressing tumor cells, or with a mixture of VEGFR2-Fc– and FGFR2-Fc–expressing tumor cells. (**a**) Renca model; (**b**) B16F10 model. Data are means ± SEM *p < 0.05 vs. Mock (Dunnett’s multiple comparison test). # and ^##^*p* < 0.05 between the indicated groups (unpaired *t*-test). (**c,d**) *In vivo* tumor growth following inoculation with mixtures of transfectants. (**c**) Renca model; (**d**) B16F10 model. Data are means ± SEM. ^#^*p* < 0.05 (unpaired *t*-test). Comparison of tumor volumes between the Mock group and Mock plus VEGFR2-Fc group was conducted using the last time point of the Mock group. *p < 0.05 (unpaired *t*-test). Comparison of tumor volumes of Mock plus VEGFR2-Fc tumors with VEGFR2-Fc plus FGFR2-Fc tumors. (**e**,**f**) Representative images of *in vivo* tumor angiogenesis following inoculation with the above mixtures of tranfectants. (**e**) Renca model; (**f**) B16F10 model. Tumor sections were stained with anti-CD31 (red) and DAPI (blue). Scale bars represent 100 μm.

To examine the roles of the FGF signaling in *in vivo* tumor growth of VEGFR2-Fc–expressing tumors, we established Renca and B16F10 tumor cells overexpressing FGFR2-Fc (the extracellular domain of mouse FGFR2 fused to the Fc region of human IgG4) (Supplementary Fig. 5a). Cell culture supernatants of the Mock and FGFR2-Fc–expressing tumor cells were collected to assess the specific binding of FGFR2-Fc to FGF1 and FGF2. FGFR2-Fc protein specifically interacted with FGF1 and FGF2, but not with VEGF (Supplementary Fig. 5b,c). Furthermore, FGFR2-Fc significantly suppressed FGF2-induced tube formation in a HUVEC sandwich tube formation assay in which HUVECs were co-cultured with FGFR2-Fc–expressing tumor cells (Supplementary Fig. 5d–g). In a similar assay where tube formation of HUVECs was induced by VEGF plus FGF2, tube formation was partially disrupted by co-culturing with either VEGFR2-Fc– or FGFR2-Fc–expressing tumor cells (Fig. 4a,b), and completely disrupted by co-culturing with a mixture of VEGFR2-Fc– and FGFR2-Fc–expressing tumor cells (Fig. 4a,b; Supplementary Fig. 6a,b). These results indicate that combination of VEGFR2-Fc and FGFR2-Fc inhibited the induction of HUVEC tube formation by VEGF plus FGF2 *in vitro*.

Next, we examined *in vivo* tumor growth and tumor angiogenesis by inoculating various combinations of Mock, VEGFR2-Fc–expressing, or FGFR2-Fc–expressing tumor cells into syngeneic mice (Fig. 4c,d) in Renca and B16F10 models. Inoculation with mixtures of Mock and VEGFR2-Fc–expressing tumor cells produced significantly slower tumor growth *in vivo* than inoculation with Mock cells alone in both models. *In vivo* tumor growth after the inoculation of mixtures of Mock and FGFR2-Fc–expressing tumor cells was similar to that of Mock cells alone in the Renca model, but was slower than that of Mock cells alone in the B16F10 model. This result suggests that *in vivo* tumor growth of the B16F10 tumors was partially dependent on the FGF signaling pathway. Inoculation with a mixture of VEGFR2-Fc– and FGFR2-Fc–expressing tumor cells showed further significant delay of *in vivo* tumor growth compared with inoculation with a mixture of Mock and VEGFR2-Fc–expressing tumor cells (Fig. 4c,d). Consistent with the results of the HUVEC tube formation assay *in vitro*, tumor angiogenesis was strongly suppressed in tumors formed from a mixture of VEGFR2-Fc– and FGFR2-Fc–expressing tumor cells compared with the other combinations above (Fig. 4e,f). The 2D *in vitro* tumor growth rates of Mock and FGFR2-Fc–expressing tumor cells were similar to each other (Supplementary Fig. 6c,d). VEGFR2-Fc specifically interacted with VEGF, and FGFR2-Fc selectively interacted with FGF1 and FGF2, in tumors expressing VEGFR2-Fc or FGFR2-Fc, respectively (Supplementary Fig. 6e,f). Taken together, these results indicate that simultaneous inhibition of both the VEGF and FGF signaling pathways in tumors caused further delay of *in vivo* tumor growth by enhancing tumor angiogenesis inhibition compared with inhibition of either VEGF or FGF signaling alone in both the Renca and B16F10 models.

### Lenvatinib inhibits tumor growth and angiogenesis in VEGFR2-Fc–expressing tumor cells

**Figure 5:**
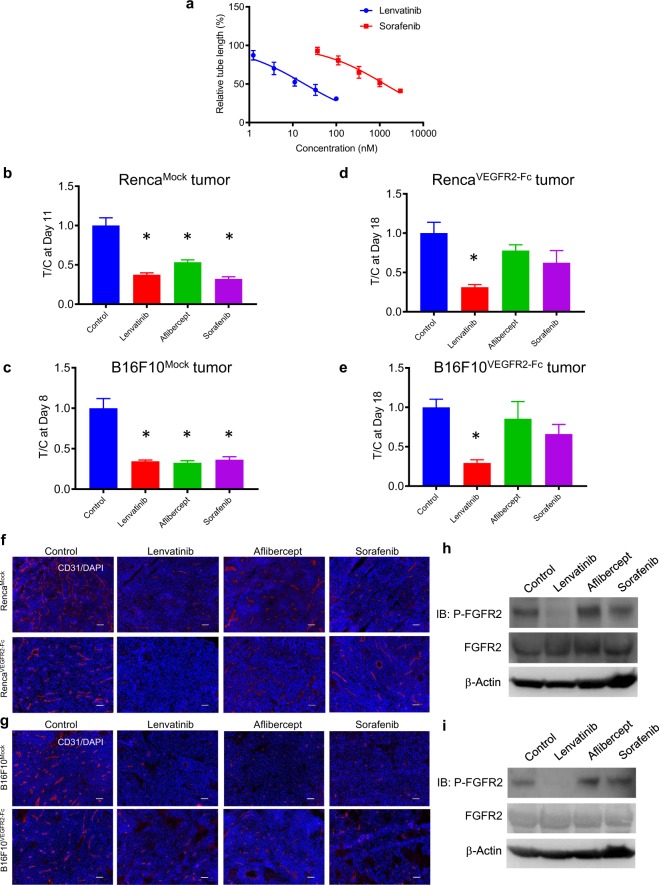
Antiangiogenesis and antitumor activity of lenvatinib, aflibercept, and sorafenib against Mock and VEGFR2-Fc–expressing tumors. (**a**) Inhibition of VEGF- plus FGF2-induced *in vitro* angiogenesis by lenvatinib and sorafenib in the HUVEC sandwich tube formation assay, measured as tube length relative to the control (non-treated) group. Data are means ± SEM (n = 6). (**b,c**) Tumor volume ratios (treatment/non-treatment control [T/C] of tumor volumes) for groups treated with lenvatinib (10 mg/kg), aflibercept (10 mg/kg), or sorafenib (30 mg/kg) in Mock tumors. (**b**) Renca model, Day 11 after treatment initiation; (**c**) B16F10 model, Day 8 after treatment initiation. Data are means ± SEM (n = 6). *p < 0.05 vs. control (Dunnett’s multiple comparison test). (**d,e**) Tumor volume ratios (treatment / non-treatment control [T/C] of tumor volumes) for groups treated with lenvatinib (10 mg/kg), aflibercept (10 mg/kg), or sorafenib (30 mg/kg) in VEGFR2-Fc–expressing tumors (same concentrations as in b, c). (**d**) Renca model and (**e**) B16F10 model at Day 18 after treatment initiation. Data are means ± SEM (n = 6). *p < 0.05 vs. control (Dunnett’s multiple comparison test). (**f,g**) *In vivo* tumor angiogenesis. Representative images of staining for CD31 (red) and DAPI (blue) in Mock and VEGFR2-Fc–expressing tumors in mice that were non-treated (control) or treated with lenvatinib, aflibercept, or sorafenib (same concentrations as (**b,c**)); tumors were resected at ~500 mm^3^. (**f**) Renca model; (**g**) B16F10 model. Scale bars represent 100 μm. (**h,i**) Western blot analysis of phosphorylation status of FGFR2 in VEGFR2-Fc–expressing tumors in mice that were non-treated (control) or treated with lenvatinib, aflibercept, or sorafenib (same concentrations as in (**b**,**c**)); tumors were resected at ~500 mm^3^. (**h**) Renca model; (**i**) B16F10 model.

Lenvatinib is a multiple tyrosine kinase inhibitor that shows dual inhibition against VEGFR and FGFR^21,22^. In contrast, sorafenib is a tyrosine kinase inhibitor that targets VEGFR, but not FGFR^23^. In a sandwich tube formation assay with HUVECs induced by VEGF plus FGF2, lenvatinib inhibited tube formation (IC_50_, 18.7 nM), and sorafenib needed higher concentrations to suppress tube formation (IC_50_, 1280 nM) (Fig. 5a). Both lenvatinib and sorafenib needed a much higher concentration (lenvatinib; IC50 > 5 μM, sorafenib; IC50 > 5 μM) to inhibit *in vitro* proliferation of Renca^Mock^, Renca^VEGFR2-Fc^, B16F10^Mock^, and B16F10^VEGFR2-Fc^ tumor cells than to suppress tube formation. Therefore, it is unlikely the *in vivo* antitumor activity of lenvatinib and sorafenib arises through direct inhibition of proliferation of cancer cells.

We next evaluated the antitumor activity of lenvatinib, aflibercept (a VEGF trap)^24,25^, and sorafenib against Mock or VEGFR2-Fc–expressing tumors in the Renca and B16F10 models. All three compounds significantly inhibited Mock tumor growth in both models (Fig. 5b,c; Supplementary Fig. 7a,b). In contrast, only lenvatinib significantly inhibited the growth of VEGFR2-Fc–expressing tumors (Fig. 5d,e and Supplementary Fig. 7c,d). To evaluate the antiangiogenic activity of lenvatinib, aflibercept, and sorafenib, resected tumors were stained with anti-CD31 Ab. Tumor angiogenesis in Mock tumors was inhibited by all three compounds in both models. In contrast, tumor angiogenesis in VEGFR2-Fc–expressing tumors was clearly inhibited by lenvatinib, although aflibercept and sorafenib appeared to slightly suppress tumor angiogenesis (Fig. 5f,g). To examine the effects that lenvatinib, aflibercept, and sorafenib on phosphorylation of FGFR2 in VEGFR-Fc–expressing tumors, we performed Western blot analysis using lysates of resected tumors. Although aflibercept and sorafenib did not decrease phosphorylation of FGFR2, lenvatinib inhibited phosphorylation of FGFR2 in both models (Fig. 5h,i). These results suggest that activation of the FGF2 signaling pathway plays a role in the escape mechanism that underlies acquired resistance to anti-VEGF therapy.

## Discussion

In this study, we demonstrated that activation of the FGF2 signaling pathway is a conserved mechanism for acquired resistance to anti-VEGF therapy in two mouse tumor models. In the VEGFR2-Fc–expressing tumors, FGF2 was expressed in pericytes immediately adjacent to the endothelial cells that expressed FGFR2. Therefore, there might be spatially effective activation of the FGF2 signaling pathway between endothelial cells and pericytes in tumor vasculatures that are resistant to anti-VEGF therapy. Because we observed that Fgfr2 mRNA was significantly and consistently induced in VEGFR2-Fc–expressing tumors in the two tumor models, we focused on this receptor. However, we also found that the mRNA levels of Fgfr3 in the Renca model and Fgfr1 and Fgfr4 in the B16F10 model were significantly upregulated by chronic expression of VEGFR2-Fc (Fig. 2c,d), consistent with previous reports that FGFR1, 3, and 4 also play a critical role in tumor angiogenesis^26,27^. Lenvatinib inhibits multiple tyrosine kinases, including VEGFR and FGFR, with similar inhibitory activity against several FGFR subtypes (FGFR1–4)^28,29^. Therefore, further analysis is required to determine the role of specific FGFR subtypes in the resistance of endothelial cells to anti-VEGF therapy.

The number of pericyte-covered vessels has been reported to increase following anti-VEGF therapy^30,31^, although the mechanism by which pericytes cover tumor vessels after such therapy remains unknown. Consistent with these reports, after long-term exposure to VEGFR2-Fc, we consistently observed that SMA^+^ cells were co-localized with CD31^+^ cells in both the Renca and B16F10 models. Because we observed fewer SMA^+^ cells in Mock tumors than VEGFR2-Fc–expressing tumors in both models, these cells, during chronic exposure to anti-VEGF therapy, might be recruited from blood into the tumor microenvironment by secreted factors or might be differentiated from other cell types in the tumor microenvironment. Indeed, mesenchymal stem cells, which derive from bone marrow, are a known source of pericytes^32,33^. Myofibroblasts^34,35^ and endothelial cells^36,37^ can also differentiate into pericytes. Therefore, to improve anti-VEGF therapy, further analyses are required to understand the underlying mechanism of the recruitment of pericytes into the tumor microenvironment or the differentiation of other cell types into pericytes, and the role of FGF expression in pericytes.

In the differential gene expression analysis, no other receptors or related ligands were consistently upregulated in both Renca and B16F10 models, but Pdgfra mRNA levels were significantly upregulated in both models. Furthermore, Angpt1 and Tek mRNA levels were increased in B16F10^VEGFR2-Fc^ tumors compared with B16F10^Mock^ tumors (Supplementary Fig. 3f). PDGF–PDGFR signaling pathways contribute to the migration of pericytes^38,39^, and the ANGPT1–TEK signaling pathway regulates maturation of blood vessels through crosstalk between pericytes and endothelial cells^40,41^. Therefore, our results suggest that tumor vessels in anti-VEGF therapy–resistant tumors are more mature than those in pre-treatment tumors. The frequency of pericyte-covered vessels increases with anti-VEGF Ab and anti-VEGFR1 Ab therapy, and it has been hypothesized that pericyte-covered vessels are resistant to anti-VEGF therapy^42–44^. The crosstalk between pericytes and endothelial cells is regulated by multiple signaling pathways, such as the EFNB2–EPHB4^45–48^, JAG1–NOTCH3^49,50^ pathways, as well as the ANGPT–TEK and PDGF –PDGFR^51,52^ pathways mentioned above. But, it is still unknown whether selective inhibition of these signaling pathways in either pericytes or endothelial cells can overcome the resistance of pericyte-covered vessels to anti-VEGF therapy. Our study reveals a new role for FGF2 in pericytes, and activation of the FGF signaling pathway in tumor vessels in anti-VEGF therapy–resistant tumors. Even after dual inhibition of VEGFR and FGFR, complete tumor growth inhibition was not achieved. To further combat resistance against antiangiogenic therapy, we might need to suppress other pro-angiogenic pathways as well: e.g., TEK, PDGFR, EPHB, and NOTCH signaling pathways. Our decoy system would be useful for evaluating the contributions of these angiogenic receptors to tumor angiogenesis.

**Figure 6:**
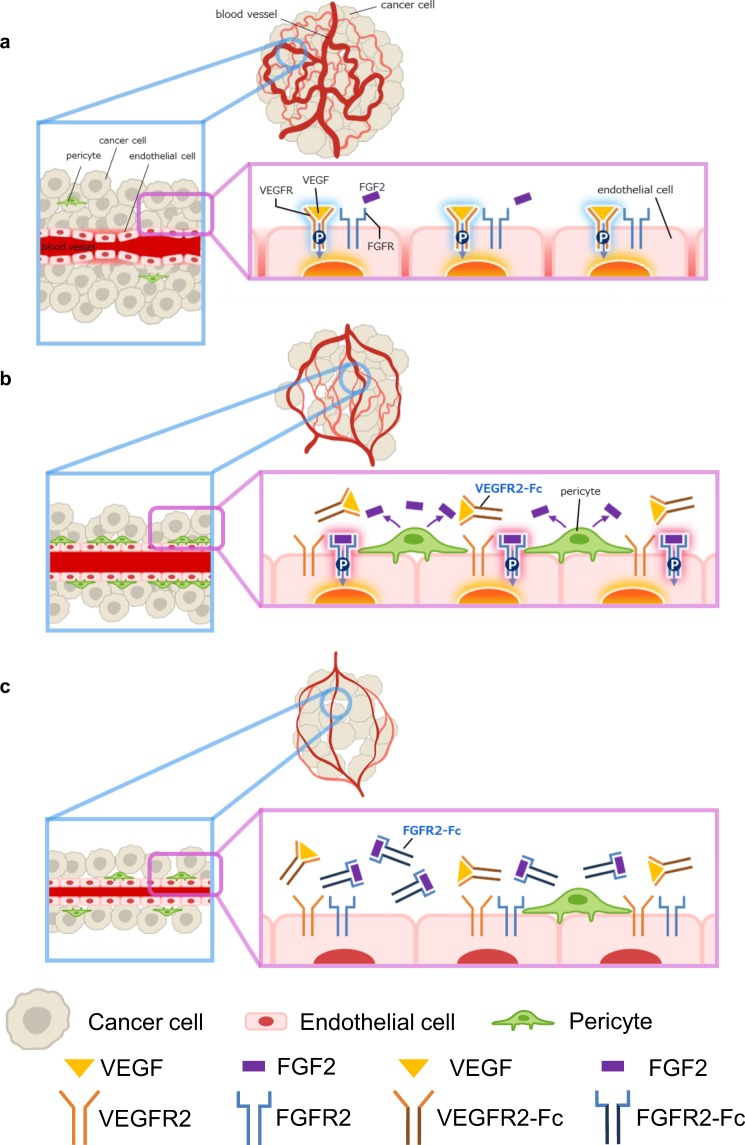
Schema of mechanisms of FGF2-driven acquired resistance to anti-VEGF therapy. (**a**) VEGF induces tumor angiogenesis by activating the VEGF signaling pathway in endothelial cells. Tumor vessels are abnormal in that they are mixtures of large and small tortuous vessels, have impaired function, and are hardly covered with pericytes. (**b**) VEGF-dependent vessel formation is suppressed by chronic expression of VEGFR2-Fc. However, the activated FGF2 signaling pathway maintains tumor vessels despite chronic inhibition of the VEGF signaling pathway. Tumor vessels covered by pericytes become resistant to VEGFR2-Fc. **c** Simultaneous expression of FGFR2-Fc and VEGFR2-Fc inhibits activation of the FGF signaling between pericytes and endothelial cells. This further decreases the number of tumor vessels and enhances tumor growth suppression. Thus, dual inhibition of FGFR and VEGFR, such as in lenvatinib treatment, enhances antitumor activity.

In summary, tumor angiogenesis mainly depends on the VEGF signaling pathway (Fig. 6a). During chronic exposure to anti-VEGF therapy, VEGF-dependent vessels are suppressed. Dependency on the FGF2 signaling pathway for tumor angiogenesis then emerges as the number of tumor vessels covered with pericytes, which overexpress FGF2, increases (Fig. 6b). Therefore, suppression of FGFR in addition to VEGFR inhibition by agents such as lenvatinib would be needed to maximize anti-VEGF therapy (Fig. 6c). Dual inhibition of VEGFR and FGFR is a mode of action unique to lenvatinib: i.e., it is different from VEGF or VEGFR selective inhibitors, antibodies against VEGF or VEGFR2, and VEGF traps. Our findings provide insight into the importance of the FGF2 signaling pathway inhibition for enhancing anti-VEGF therapy, thus improving antiangiogenic therapy.

## Methods

### Plasmid construction

Mouse VEGFR2 extracellular domain (amino acid residues 20–762; GenBank ID, BC020530), FGFR2 extracellular domain (residues 22–367; RefSeq accession number, NM_010207.2), and the human IGHG4 Fc region (GenBank ID, BC025985.1) were subcloned into PB510B vector (System Biosciences) to produce vectors expressing VEGFR2-Fc, FGFR2-Fc, and soluble Fc, respectively. The signal peptide of VEGFR2 (residues 1–19) and FGFR2 (residues 1–22) were replaced by that of human IL-2 (residues 1–20) to enable effective secretion.

### Western blot analysis

Cell lysates were prepared with lysis buffer (Cell Signaling Technology). Proteins were separated by SDS-PAGE and immunoblotted with the indicated antibodies. Digital images were captured by using the LAS-3000 Imaging System (Fujifilm). The following were used as primary antibodies for Western blot analysis: VEGF (R&D Systems #AF-493-NA; 1:1000); FGFR2 (Thermo Fisher Scientific #PA5-14651; 1:1000) and Phospho-FGFR2 (Thermo Fisher Scientific #PA5-64796; 1:1000); IGHG4 (abcam #ab99820; 1:1000), FGF1 (abcam #ab169748; 1:1000), and VEGFR2 (abcam #ab11939; 1:1000); FGF2 (Santa Cruz Biotechnology #sc271847; 1:1000); and β-Actin (Sigma #A5441; 1:5000). Anti-goat IgG HRP (horseradish peroxidase) (abcam #ab97110, 1:5000); anti-rabbit IgG HRP (Cell Signaling Technology #7074; 1:5000) and anti-mouse IgG HRP (Cell Signaling Technology #7076; 1:5000) were used as secondary antibodies.

### Immunoprecipitation assay

Cell culture supernatants of Mock, VEGFR2-Fc, FGFR2-Fc, and soluble Fc transfectants were collected in separate Eppendorf tubes. Protein A beads and recombinant proteins (VEGF, FGF1, or FGF2 [R&D systems]) were added to the tubes and rotated overnight at 4 °C. For *in vivo* immunoprecipitation assays, tumor lysates were prepared from resected tumor tissues. To immunoprecipitate endogenous VEGF, FGF1, and FGF2, protein A beads alone were added to the tubes. Precipitates were washed with lysate buffer. All samples were added to SDS sample buffer, boiled at 95 °C, and assessed by Western blotting.

### RT-qPCR analysis

Tumor tissues were resected at volumes of ~500 mm^3^ and total RNA was prepared by Maxwell system (Promega). Isolated RNA was used for RT-qPCR using TaqMan Fast Universal PCR Master Mix and TaqMan gene expression assay kits (Applied Biosystems/Life Technologies). The following gene-specific TaqMan gene expression assay kits were used: Fgfr1, Mm01215485_g1; Fgfr2, Mm01269930_m1; Fgfr3, Mm00433294_m1; Fgfr4, Mm01341852_m1; Fgf1, Mm01258325_m1; Fgf2, Mm01285715_m1; Pecam1, Mm01242576_m1; Acta2, Mm00725412_s1; Ptprc, Mm01293577_m1; and Actb, Mm04394036_g1. Expression levels were normalized to the internal control (Actb). Relative expression levels were calculated as the normalized mRNA level compared with the Mock control.

### *In vitro* cell growth assay

Transfectants were plated on 96-well plates at 0.5 to 2 × 10^3^ cells/well and cultured at 37 °C under a 5% CO_2_ atmosphere. To test the antiproliferative activity of compounds, the cells were treated with lenvatinib or sorafenib (0.01–10 μM) for 3 days. Numbers of cells were determined by using a Cell Counting Kit-8 (Dojindo) or Cell Titer-Glo (Promega).

### Sandwich tube formation assay

HUVECs were isolated from a human umbilical cord that was obtained with informed consent in the Shoji clinic (Tsukuba, Japan). The experiments using the umbilical cord and isolated HUVECs were approved by the Research Ethics Committee of Eisai Co., Ltd. All experiments were performed in accordance with the guidelines. HUVECs were maintained at subconfluence by changing the culture medium every day. An aliquot (0.4 mL) of the collagen solution (Nitta Gelatin) was added to each well of 24-well plates and allowed to form gel. HUVECs were harvested by trypsin–EDTA, plated onto the gel at 1.5 × 10^5^ cells per well with serum-free medium (Invitrogen) containing EGF (Invitrogen), and incubated overnight. Medium was removed, and 0.4 mL of the collagen solution was added to the cells and allowed to form gel for 4 h at 37 °C. An aliquot of 1 mL of assay medium plus VEGF and/or FGF2 containing Renca or B16F10 transfectants were added to each well. HUVECs sandwiched in collagen gel were incubated for 2–4 days at 37 °C. The medium was removed, and 0.4 mL of MTT solution (Sigma) was added to each well and incubated for another 4 h. Images were taken with a light microscope. Tube length was measured using an Angiogenesis Image Analyzer (Kurabo)^53^. For evaluation of lenvatinib and sorafenib, an aliquot of 1 mL of assay medium plus VEGF or FGF2 and lenvatinib or sorafenib was added to each sandwiched well. IC_50_ values were determined using GraphPad Prism version 7.02 (GraphPad Software).

### Animal experiments

All animal experiments were approved by the Institutional Animal Care and Use Committee of Eisai. and conducted in accordance with the guidelines for animal experiments of the Institutional Animal Care and Use Committee of Eisai. Total of 2 × 10^6^ cells of either one transfectant or a mixture of two transfectants was subcutaneously inoculated into the right flanks of female BALB/cAnNCrlCrlj mice for the Renca model or female C57BL/6 J mice for the B16F10 model. Day 1 indicates the next day after inoculation of tumor cells into the mice. Tumor volume was measured 1–3 times per week. To test the antitumor activities of lenvatinib, aflibercept, and sorafenib, mice were subcutaneously inoculated with Mock or VEGFR2-Fc–expressing cells for tumor development. Day 1 indicates the point when the tumor volume average reached ~300 mm^3^. Mice were allocated randomly into each group (Day1). Lenvatinib and sorafenib were orally administered to the tumor-bearing mice once daily for 18 days. Aflibercept (Santen Pharmaceutical Co., Ltd) was intraperitoneally injected twice a week. All groups consisted of 6 to 10 mice.

### Immunofluorescence staining analysis

For immunofluorescence staining, resected tumors were collected at volumes of ~500 mm^3^, embedded and frozen in OCT compound (Sakura Finetek). Tumor blocks were cut into 10-μm sections using a cryostat (Leica Biosystems). Tumor sections on microscope slides were fixed in methanol for 10 min. After washing with PBS, the sections were permeabilized and incubated with PBS containing 1% BSA and 0.1% Tween 20 for 1h. Tumor sections were incubated with anti-CD31 Ab (BD Pharmingen #553370; 1:200), anti-FGF2 Ab (Thermo Fisher Scientific #PA1-18362; 1:200), and anti-SMA Ab (Abcam #ab5694; 1:200) at 4 °C overnight. The sections were washed three times and incubated with appropriate secondary antibodies and DAPI (Dojindo #340-07971; 1:500) for 30 min. Stained sections were washed with PBS and mounted in Prolong Gold Antifade Reagent (Thermo Fisher Scientific). Fluorescence microscopic images were captured by using a BZ-X710 microscope (KEYENCE).

### RNA-Seq analysis

Tumor tissues were resected at volumes of ~500 mm^3^. Total RNA samples were extracted by using the Maxwell Total RNA Purification kit, and RNA-Seq was performed at the GENEWIZ (HI-SEQ) next-generation sequencing platform. Raw reads for individual samples were assessed with the FastQC program (http://www.bioinformatics.babraham.ac.uk/projects/fastqc/) to check sequence quality and processed with Trimmomatic version 0.36^54^ to remove adaptor sequences and bases with low quality scores. Filtered reads were mapped to the mouse genome (Mm10 & Ensembl 88) by STAR version 2.5.2b^55^. The number of reads mapped to each gene was counted by htseq-count version 0.6.1^56^. Gene expression levels were quantified as transcripts per million using RSEM version 1.2.31^57^, and relative gene expression levels were calculated as the ratio to the mean of all Mock samples. Gene expression analysis was performed by Strand NGS software (Agilent technology Inc.) using the gene-level read counts as input. Genes that were differentially expressed between two conditions (VEGFR2-Fc vs. Mock) were identified according to two criteria: (1) fold change and (2) statistical significance determined by unpaired Student’s *t*-test followed by Benjamini–Hochberg correction. Note that we set different criteria to identify differential expressed genes: fold change >1.5 and p-value < 0.05 in Renca; fold change >2.0 and p-value < 0.001 in B16F10. Activated pathways were enumerated by uploading the differentially expressed genes to Reactome pathway analysis via the web-user interface (https://reactome.org/PathwayBrowser/#TOOL = AT)^58^.

### Isolation of endothelial cell, pericytes, and tumor-infiltrating lymphocytes

Tumors were resected at volumes of ~500 mm3, placed into Tumor Dissociation Kit reagent (Miltenyi Biotech), and dissociated by using a gentleMACS Dissociator (Miltenyi Biotec). To isolate endothelial cells, pericytes, and tumor-infiltrating lymphocytes, single-cell suspensions in MACS buffer were mixed with CD31 beads, Thy-1 beads, or CD45 beads, respectively (all from Miltenyi Biotec) and incubated for 15 min on ice. These various cell types were then isolated by using an OctoMACS separator (Miltenyi Biotec). All procedures were performed according to the manufacturer’s instructions. Total RNA was purified by using a Maxwell system (Promega), and RT-qPCR analysis was performed to determine mRNA expression levels.

### Statistical analysis

All data are presented as means ± standard error of the mean (SEM). The differences between the means of groups were analyzed by unpaired Student’s *t*-test or by one-way analysis of variance (ANOVA) followed by Dunnett’s multiple comparison test. *P* values less than 0.05 were considered statistically significant. Statistical analyses were performed using GraphPad Prism version 7.02.

## Supplementary information

Supplementary information.
