# Supplementary material for: Activated FGF2 signaling pathway in tumor vasculature is essential for acquired resistance to anti-VEGF therapy
Source: Sci Rep. 2020 Feb 19;10:2939. doi: 10.1038/s41598-020-59853-z (PMC7031295; doi:10.1038/s41598-020-59853-z)

Activated FGF2 signaling pathway in tumor vasculature is essential for acquired resistance to anti-VEGF therapy

Kenji Ichikawa*, Saori Watanabe Miyano, Yukinori Minoshima, Junji Matsui, & Yasuhiro Funahashi*

Affiliation

Tsukuba Research Laboratories, Eisai Co., Ltd., Tsukuba, Ibaraki

5-1-3 Tokodai, Tsukuba, Ibaraki 300-2635, Japan

Correspondence and requests for materials should be addressed to Kenji Ichikawa (k2-ichikawa@hhc.eisai.co.jp) and Yasuhiro Funahashi (y-funahashi@hhc.eisai.co.jp)

Supplementary Fig.1

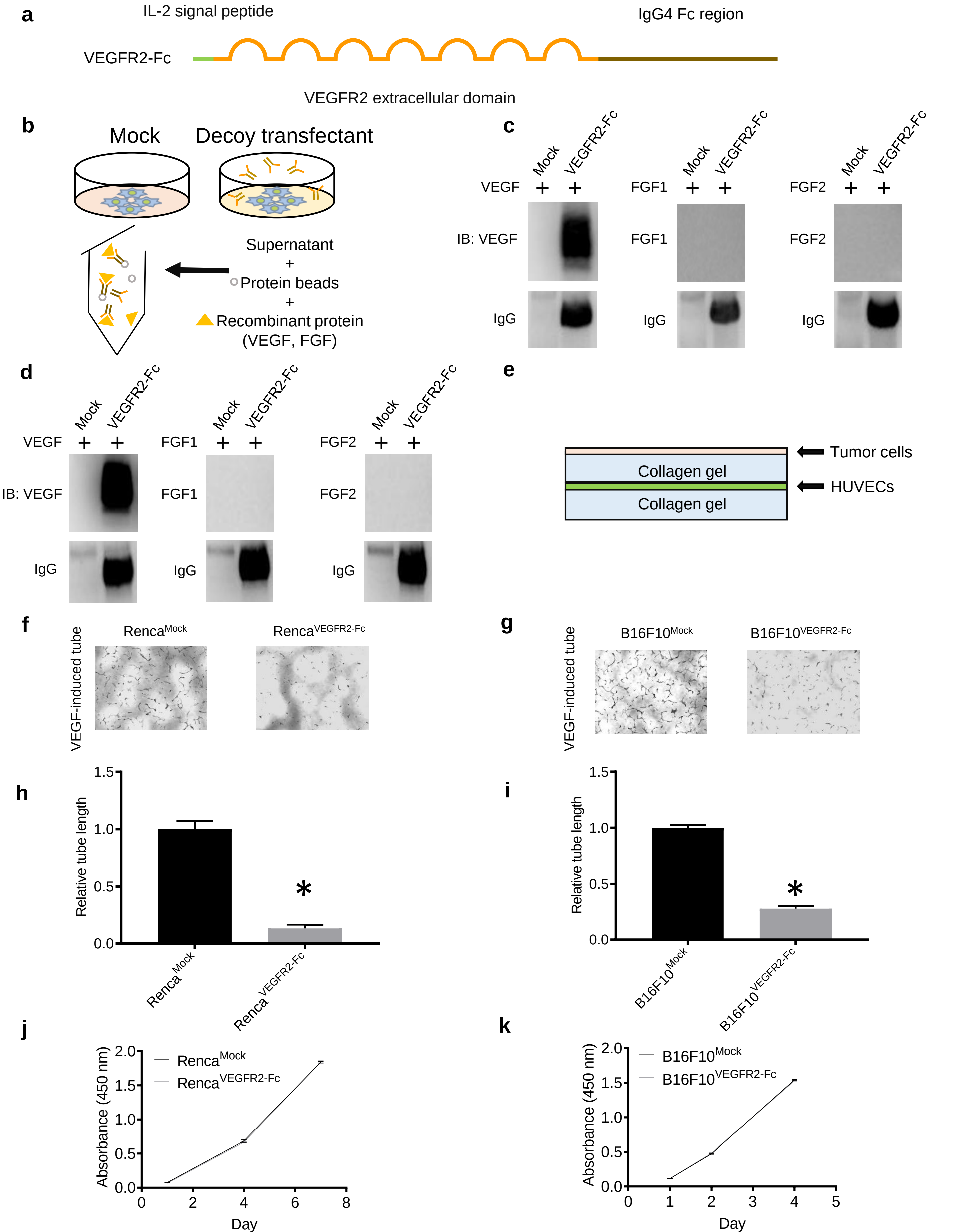

Supplementary Fig.1

Supplementary Fig. 1 Functional analysis of VEGFR2-Fc in Renca and B16F10 cells *in vitro*.

a Schematic of VEGFR2-Fc.

b Immunoprecipitation assay for VEGF and FGF recombinant proteins.

c, d Co-precipitation of VEGF, FGF1, or FGF2 recombinant protein with VEGFR2-Fc from culture supernatants. Immunoprecipitated VEGF, FGF1, and FGF2 with VEGFR2-Fc were detected with the indicated antibodies. **c**, Renca model; **d**, B16F10 model.

e Schematic of HUVEC sandwich tube formation in co-cultures with tumor cells.

f, g Representative images of HUVEC sandwich tube formation induced by VEGF. **f**, Renca model; **g**, B16F10 model.

h, i Inhibition of HUVEC sandwich tube formation induced by VEGF in co-cultures with **h** $Renca^{Mock}$ or $Renca^{VEGFR2\text{-}Fc}$ cells and **i** $B16F10^{Mock}$ or $B16F10^{VEGFR2\text{-}Fc}$ cells. Tube lengths were measured relative to those with the Mock cells. Data are means ± SEM (n = 6). $^*p < 0.05$ vs. Mock control (unpaired *t*-test).

j, k *In vitro* 2D growth assays of Mock and VEGFR2-Fc–expressing tumor cells. **j**, Renca model; **k**, B16F10 model.

Supplementary Fig.2

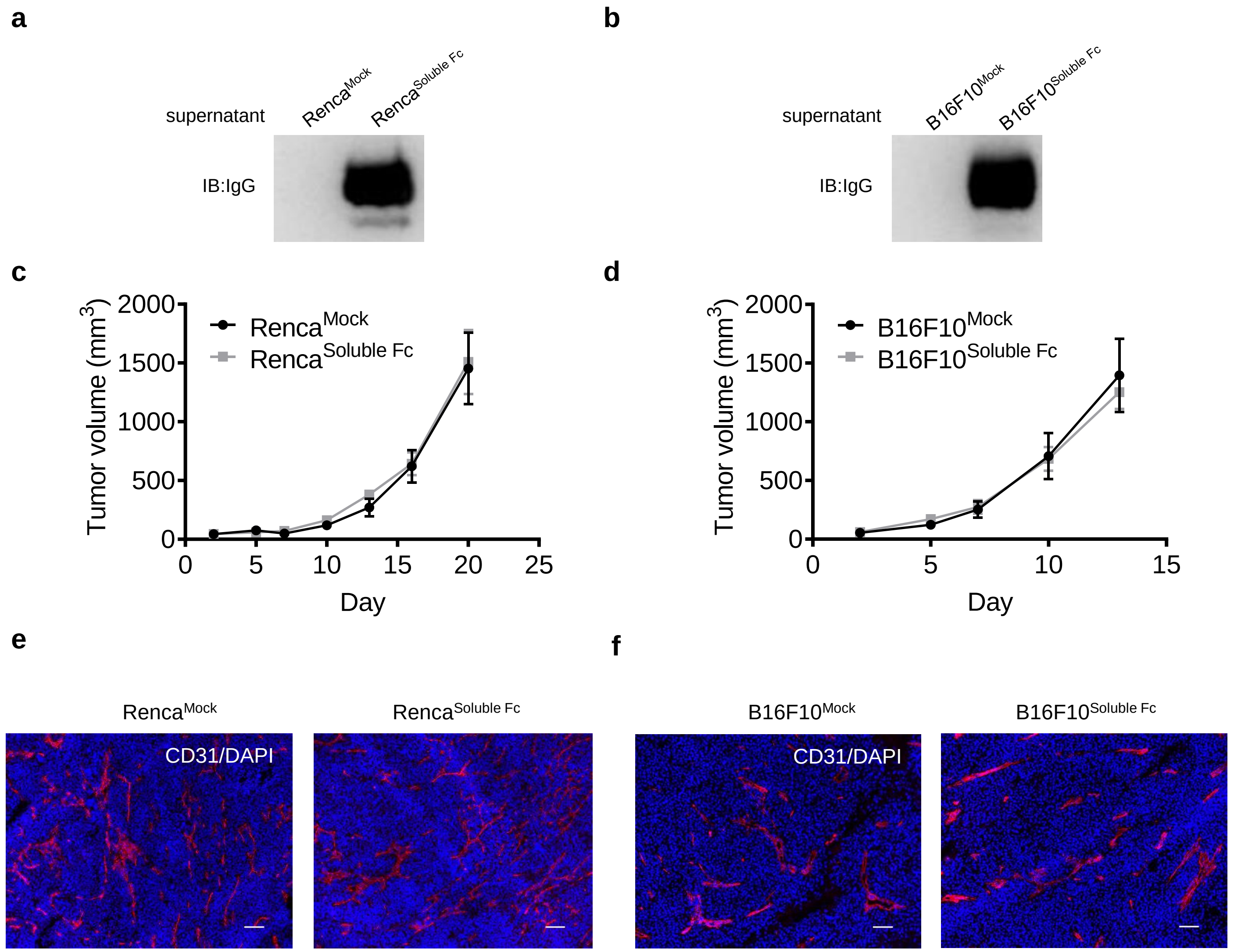

Supplementary Fig. 2 *In vivo* tumor growth and tumor angiogenesis of soluble Fc–expressing tumors.

a, b Secretion of soluble Fc proteins into the culture supernatants. **a,** Renca model; **b,** B16F10 model.

c, d *In vivo* tumor growth of Mock and soluble Fc–expressing tumors. **c**, Renca model; **d**, B16F10 model. Data are means ± SEM (n = 6)

e, f Representative images of CD31 staining (endothelial cell marker) in **e** Renca^{Mock} and Renca$^{soluble\ Fc}$ tumors and **f** B16F10^{Mock} and B16F10$^{soluble\ Fc}$ tumors. Scale bars represent 100 μm.

Supplementary Fig.3

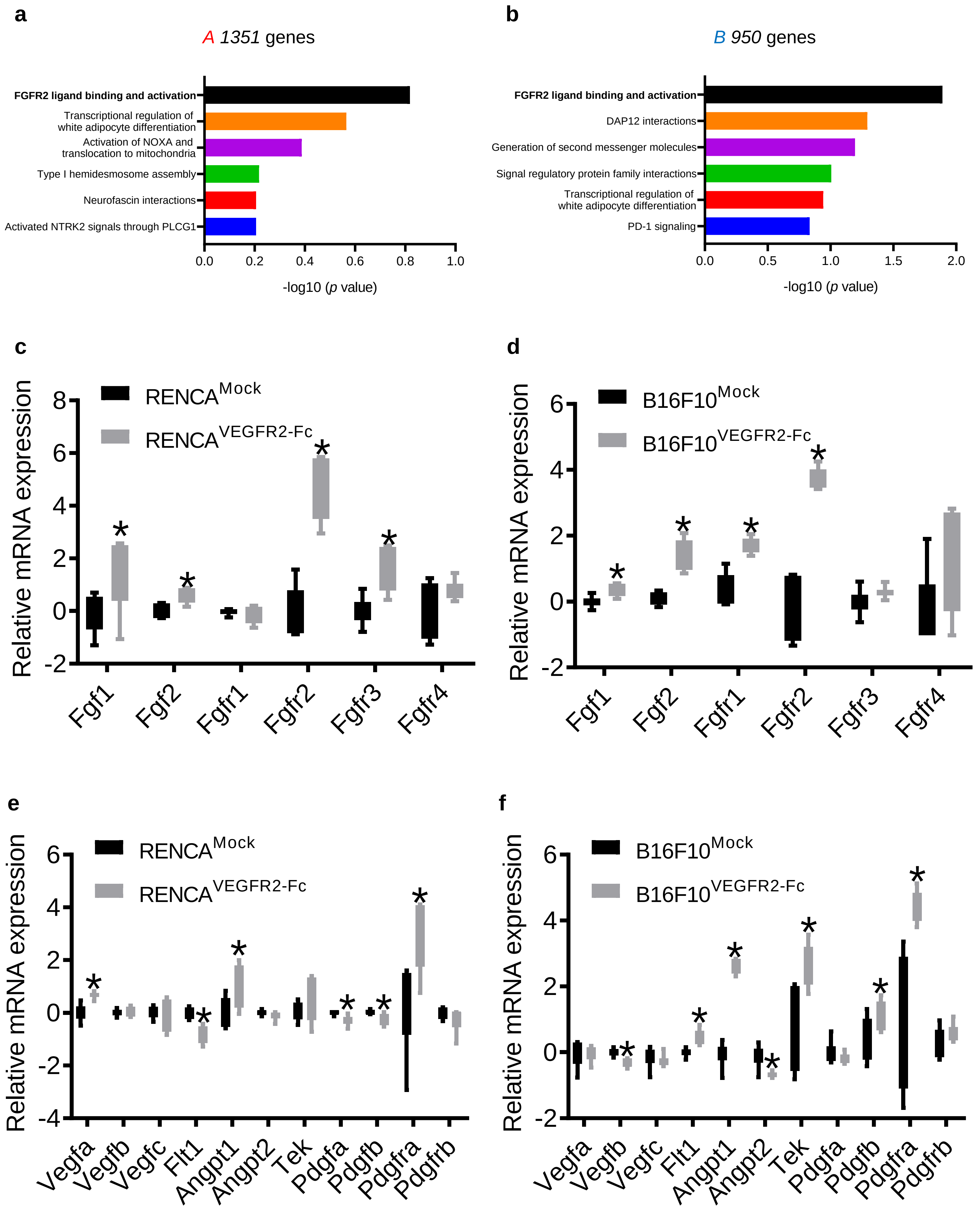

Supplementary Fig.3 (continued)

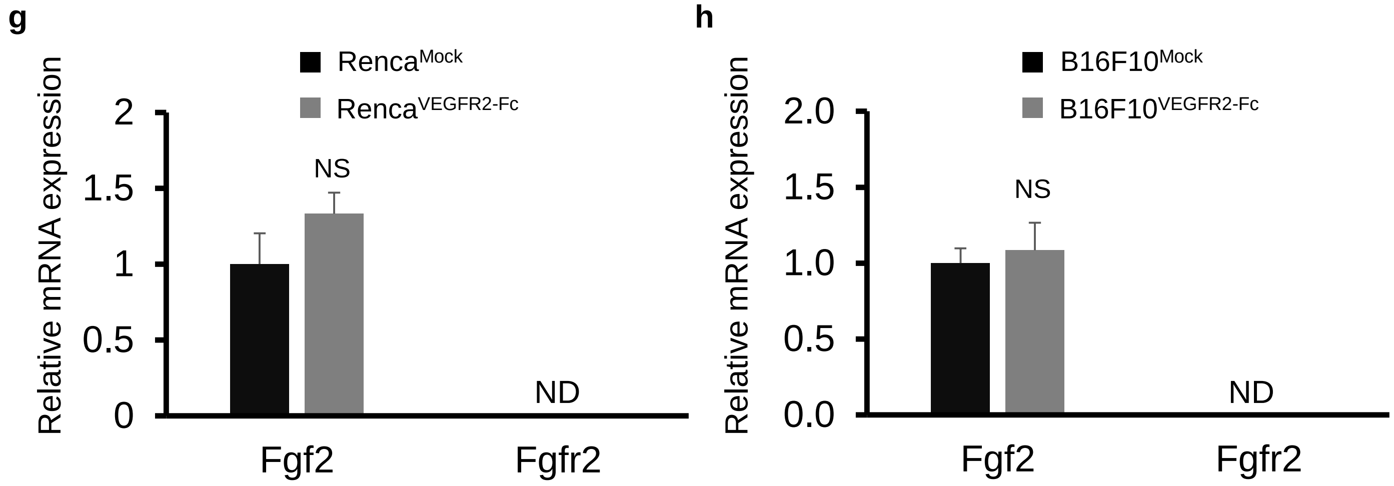

Supplementary Fig. 3 Pathway analysis of genes differentially expressed in VEGFR2-Fc–expressing tumors compared with Mock tumors in Renca and B16F10 models.

a, b Reactome analysis of signaling pathway using genes upregulated (>1.5-fold) in VEGFR2-Fc–expressing tumors. **a**, Renca model; **b**, B16F10 model. The top 6 pathways are shown.

c–f RNA-seq analysis of mRNA levels in VEGFR2-Fc–expressing tumors. **c**, **d**, FGF and FGFR family members. **c**, Renca model; **d**, B16F10 model. **e**, **f**, Other pro-angiogenic factors. **e**, Renca model; **f**, B16F10 model. Boxplots show log2-transformed fold change relative to Mock samples for selected genes (n = 6). *$P < 0.05$ (unpaired Student's *t*-test).

g, h RT-qPCR analysis of the mRNA levels of Fgf2 and Fgfr2 in cultured cells. **g**, $\text{Renca}^{\text{Mock}}$ and $\text{Renca}^{\text{VEGFR2-Fc}}$ cells; **h**, $\text{B16F10}^{\text{Mock}}$ and $\text{B16F10}^{\text{VEGFR2-Fc}}$ cells. Data are means ± SEM (n = 6). *$P < 0.05$ (unpaired Student's *t*-test). NS, not significant. ND, not detected.

Supplementary Fig.4

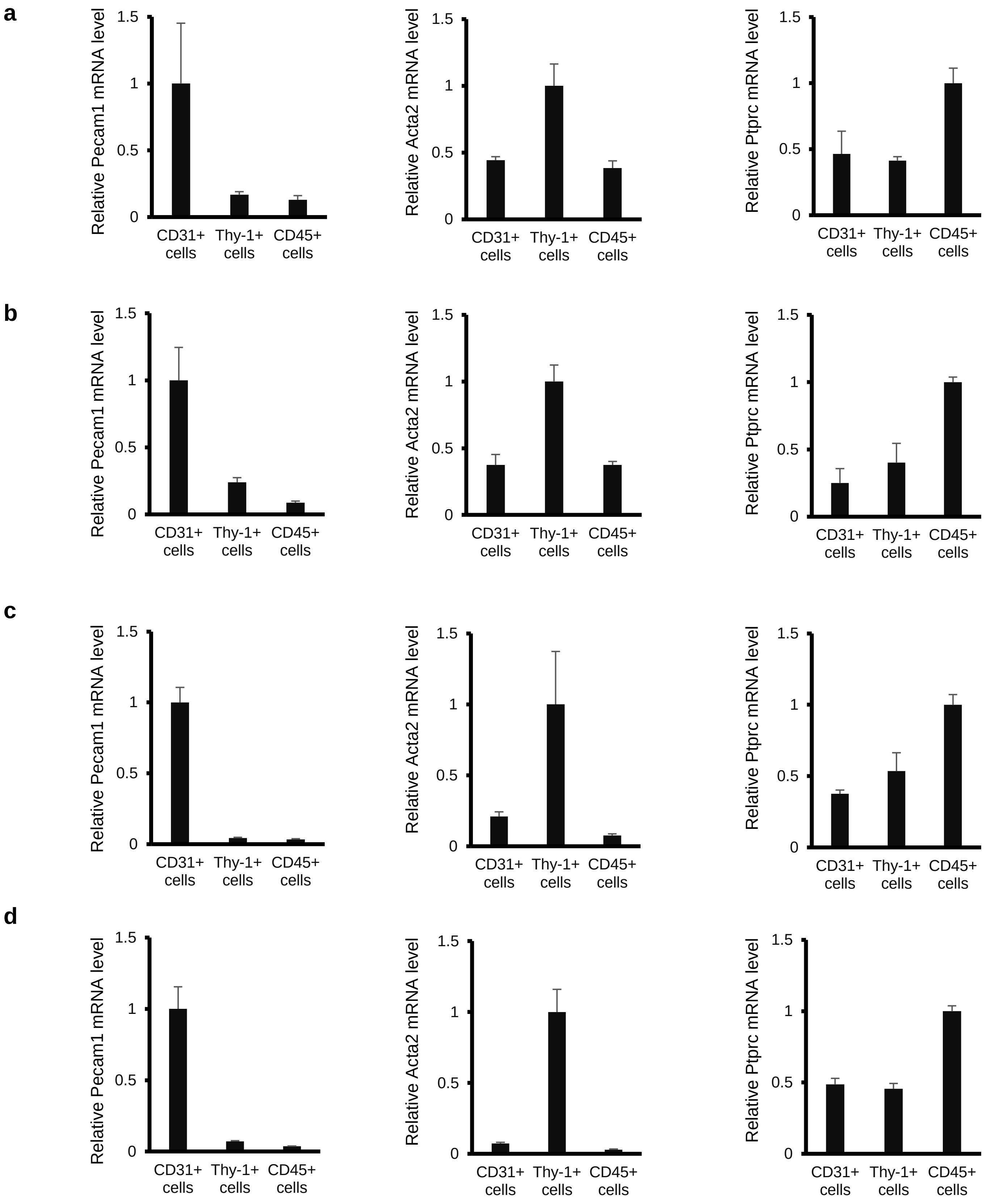

Supplementary Fig.4

Supplementary Fig. 4 RT-qPCR analysis of mRNA expressions of Pecam1, Acta2, and Ptpcr in isolated cells by MACS beads.

a–d Pecam1, Acta2, and Ptpcr mRNA expression in cells isolated with anti-CD31, Thy-1, and CD45 Ab-coated microbeads analysis. **a**, $Renca^{Mock}$; **b**, $Renca^{VEGFR2\text{-}Fc}$; **c**, $B16F10^{Mock}$; **d**, $B16F10^{VEGFR2\text{-}Fc}$ tumors. In each panel, mRNA expression levels are shown relative to the expression of positive controls (left panel, $CD31^+$ cells; middle panel, Thy-1^+ cells; right panel, $CD45^+$ cells). Data are means ± SEM (n = 5).

Supplementary Fig.5

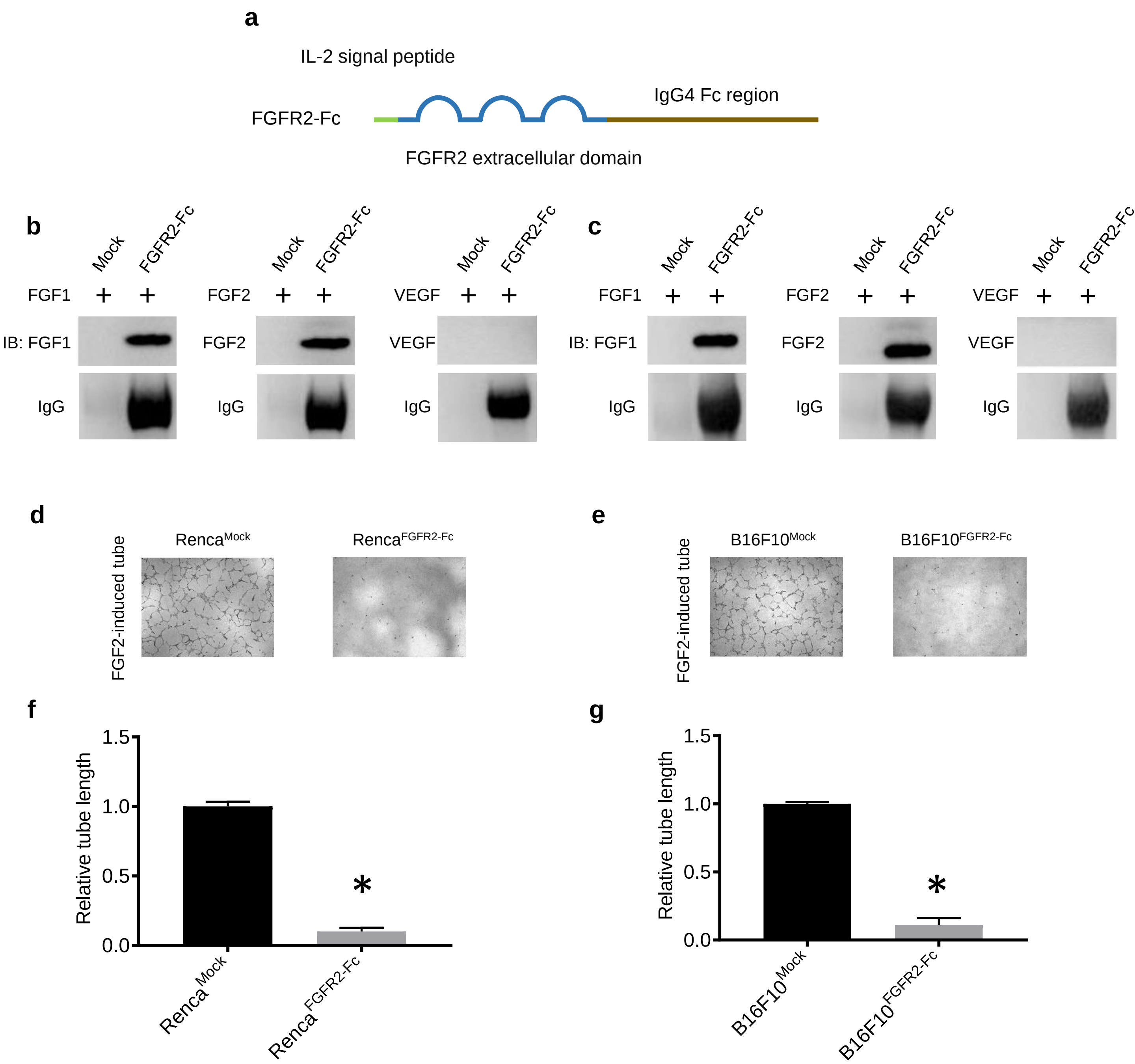

Supplementary Fig. 5 Functional analysis of FGFR2-Fc in Renca and B16F10 cells *in vitro*.

a Schematic of FGFR2-Fc.

b, c Co-precipitation of FGF1, FGF2, or VEGF recombinant protein with FGFR2-Fc from culture supernatants. Immunoprecipitated FGF1, FGF2, and VEGF with FGFR2-Fc were detected with the indicated antibodies **b**, Renca model; **c**, B16F10 model.

d, e Representative images of HUVEC sandwich tube formation induced by FGF2. **d**, Renca model; **e**, B16F10 model.

f, g Inhibitions of HUVEC sandwich tube formation induced by FGF2 in co-cultures with **f** $Renca^{Mock}$ or $Renca^{FGFR2\text{-}Fc}$ and **g** $B16F10^{Mock}$ or $B16F10^{FGFR2\text{-}Fc}$. Data are means ± SEM (n = 6). *, $P < 0.05$ (unpaired Student's t-test)

Supplementary Fig.6

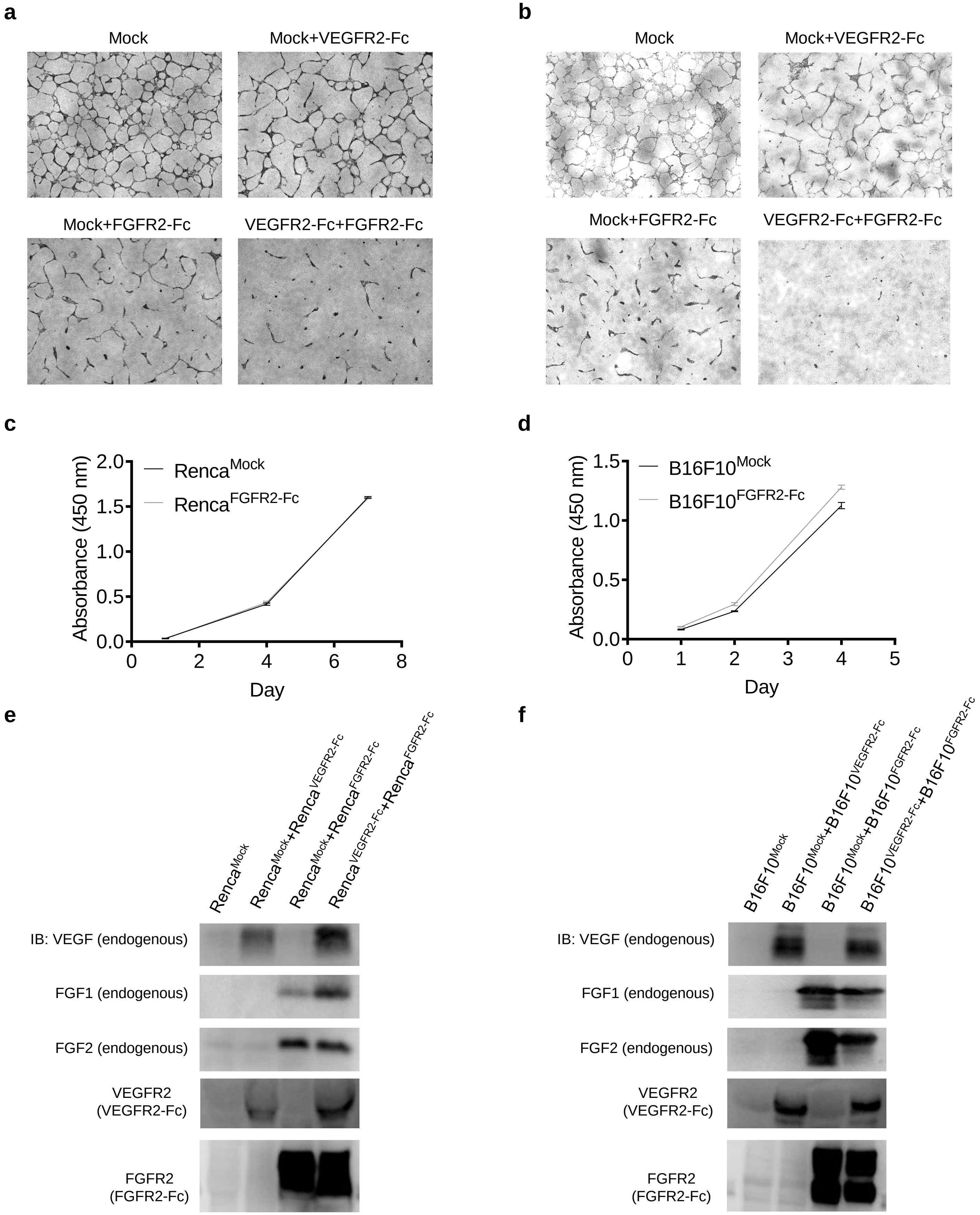

Supplementary Fig.6

Supplementary Fig. 6 Effects of VEGFR2-Fc plus FGFR2-Fc on HUVEC tube formation and tumor growth.

a, b Representative images of HUVEC sandwich tube formation induced by VEGF plus FGF2. **a,** Renca model; **b,** B16F10 model.

c, d Evaluation of 2D growth ratios between Mock and FGFR2-Fc–expressing cells. **c,** Renca model; **d,** B16F10 model.

e, f Immunoprecipitation assay for co-precipitation of VEGFR2-Fc and FGFR2-Fc with endogenous VEGF, FGF1, and FGF2 in tumor lysates. **e,** Renca model; **f,** B16F10 model.

Supplementary Fig.7

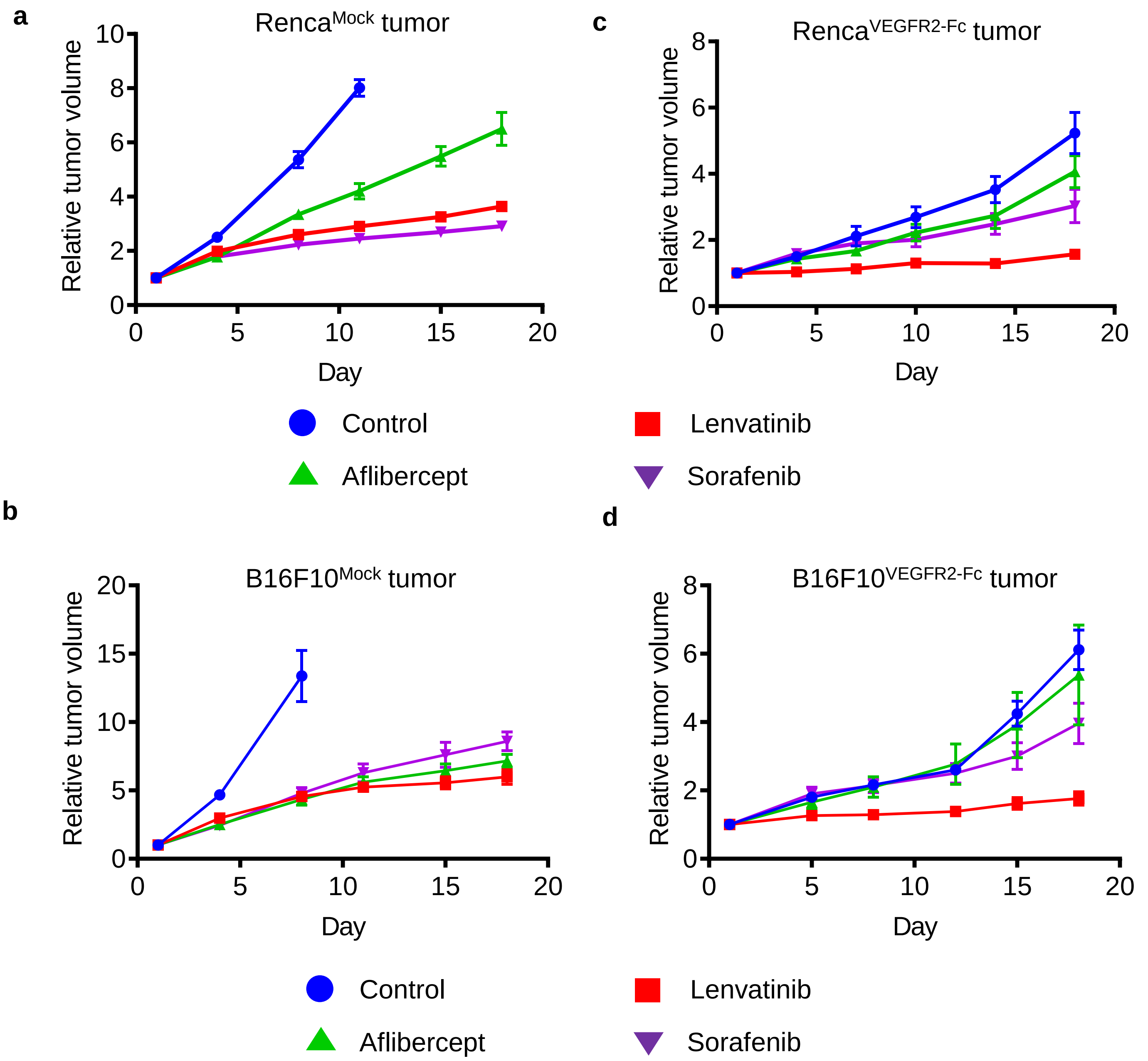

Supplementary Fig. 7 Antitumor activity of lenvatinib, aflibercept, and sorafenib against Mock and VEGFR2-Fc-expressing tumors

a–d Relative tumor volumes of Mock and VEGFR2-Fc expressing tumors in mice treated with lenvatinib 10mg/kg, aflibercept 10mg/kg, or sorafenib 30mg/kg. Treatments started at tumor sizes of ~300 mm^3 (Day 1). **a**, Renca^{Mock}; **b**, B16F10^{Mock}; **c**, Renca$^{VEGFR2\text{-}Fc}$; **d**, B16F10$^{VEGFR2\text{-}Fc}$ tumors. Data are means ± SEM (n = 6).

Supplementary Fig.8 (Full size blots)

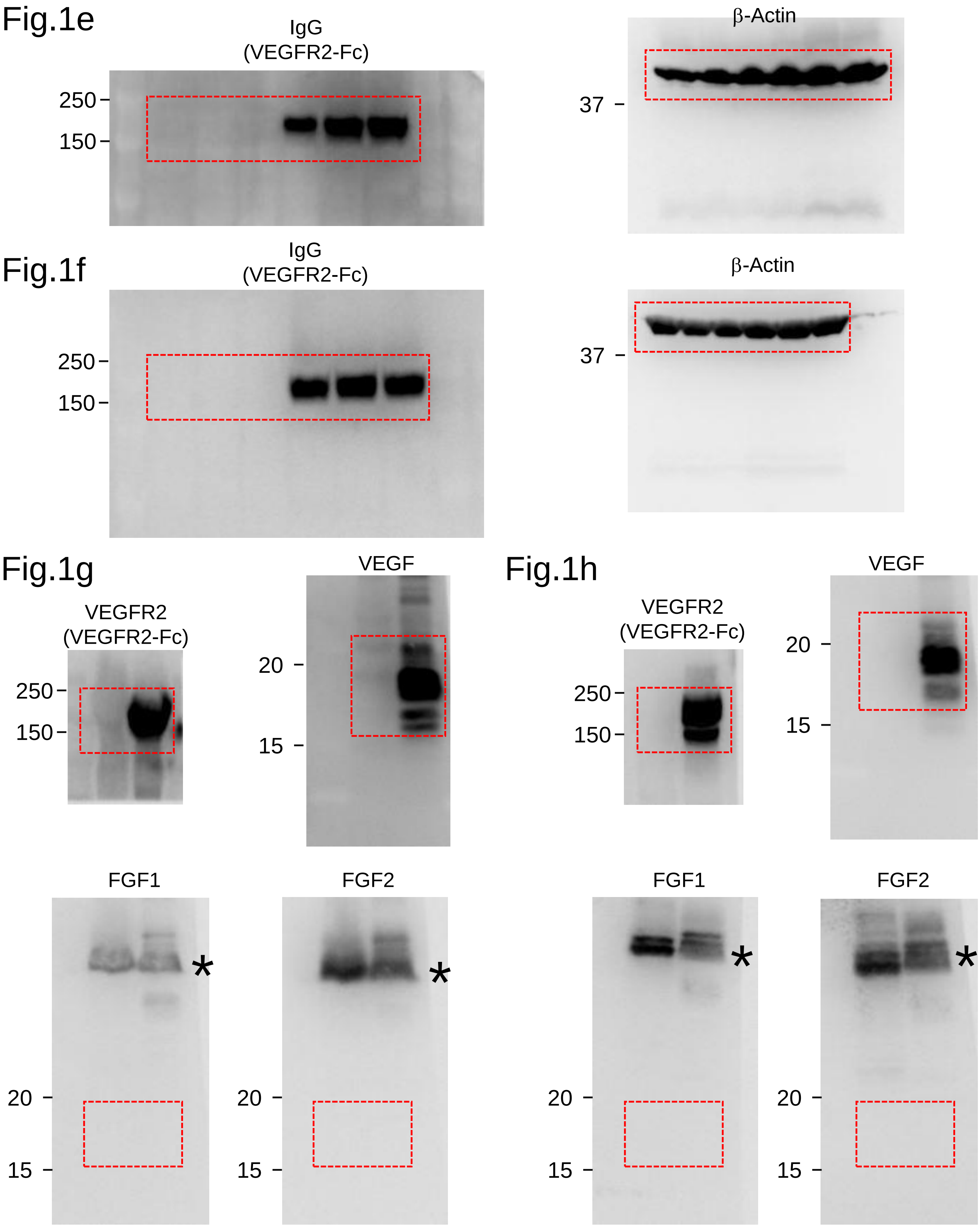

*: non-specific band

Supplementary Fig.8 (continued)

Fig.2e

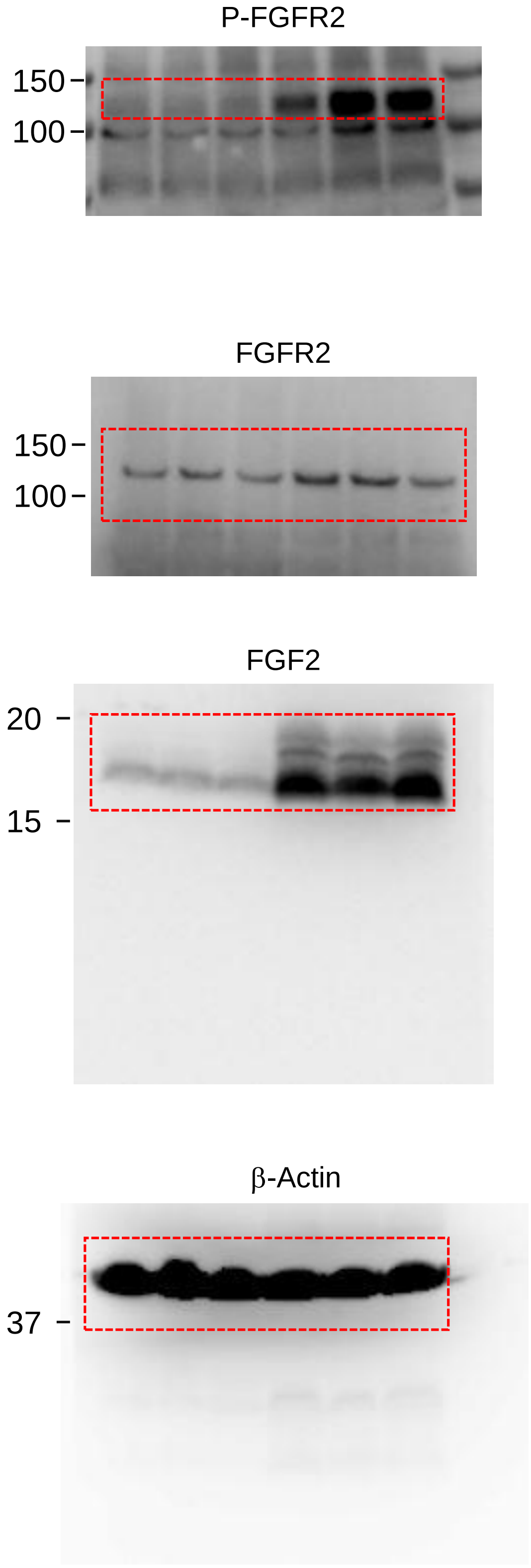

Fig.2f

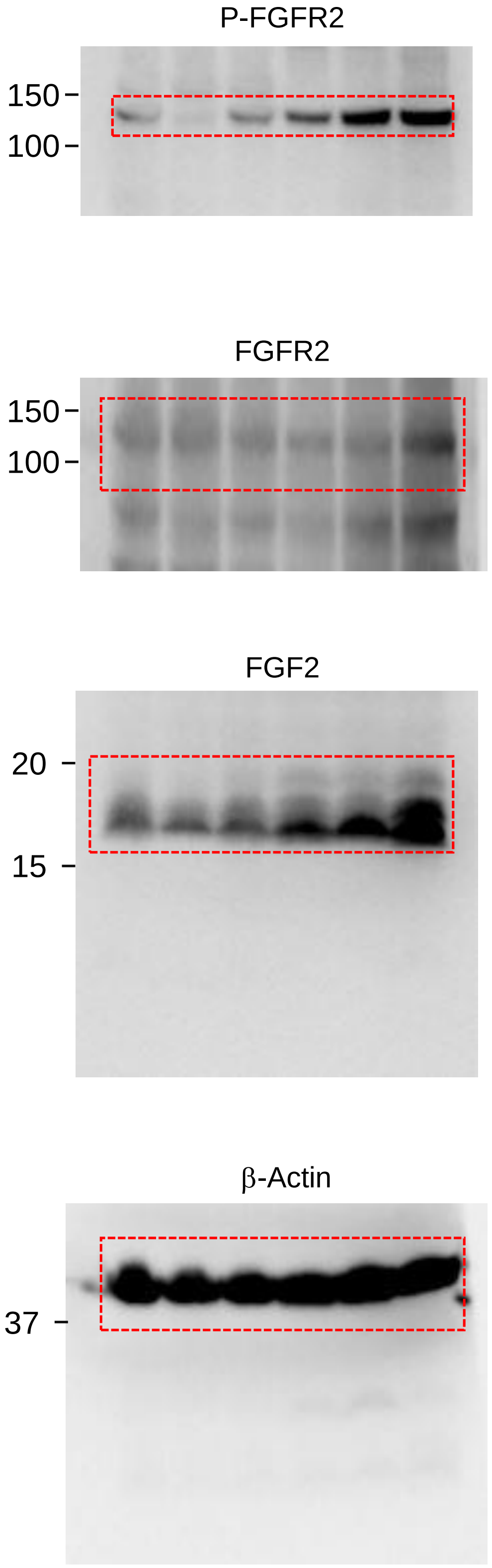

Supplementary Fig.8 (continued)

Fig.5h

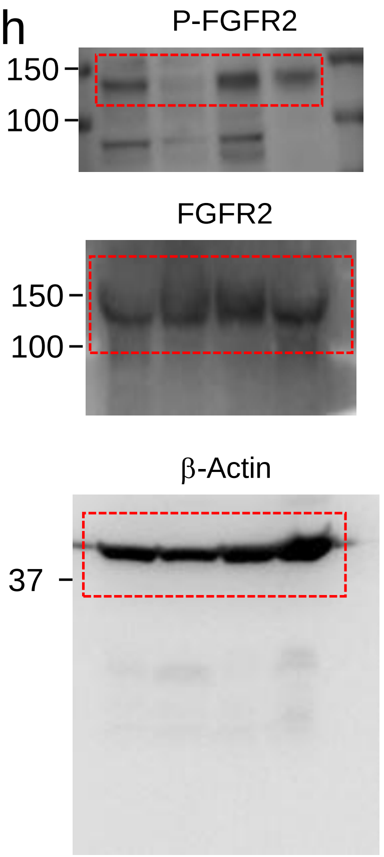

Fig.5i

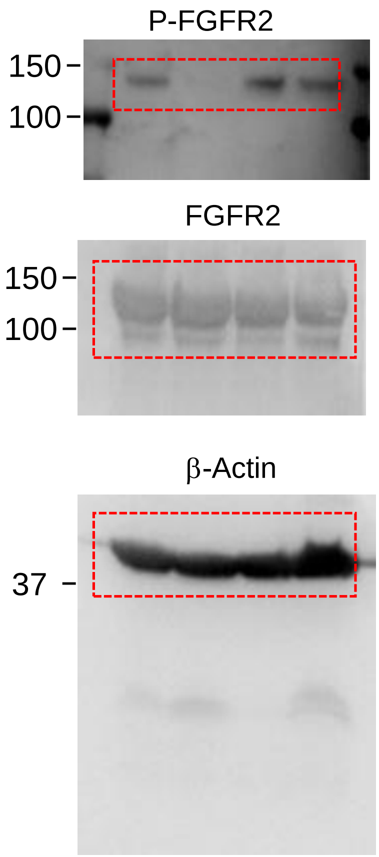

Supplementary Fig.1c

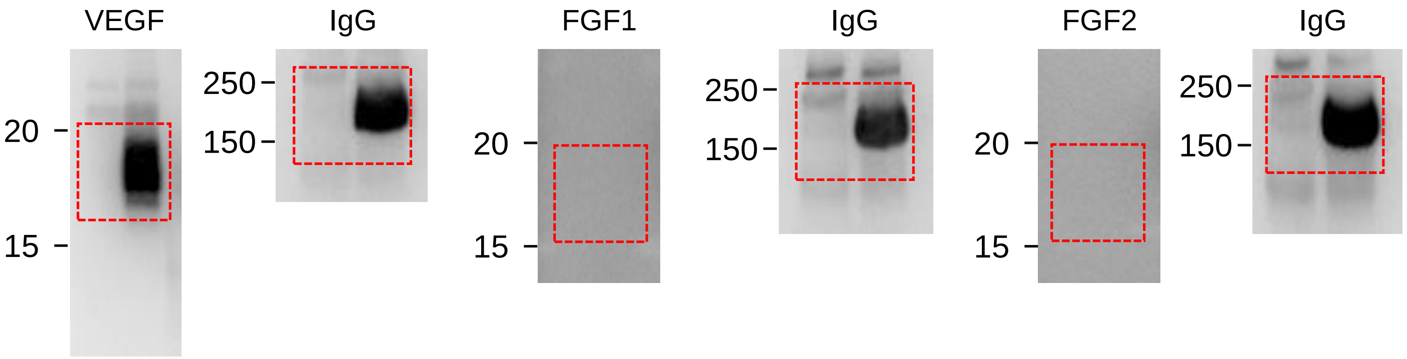

Supplementary Fig.1d

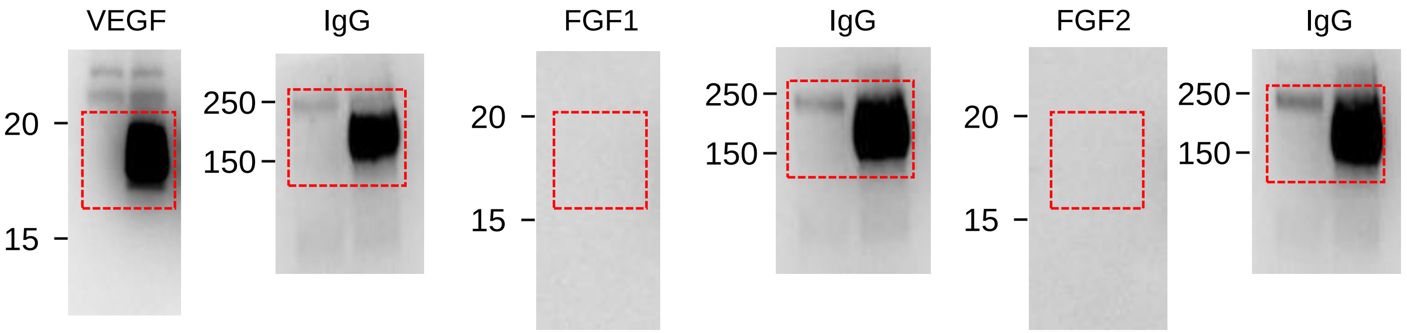

Supplementary Fig.8 (continued)

Supplementary Fig.2a

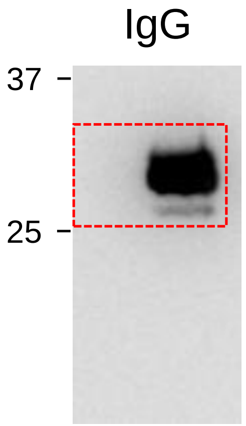

Supplementary Fig.2b

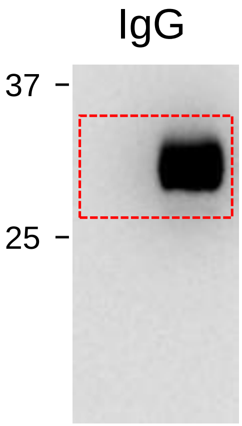

Supplementary Fig.5b

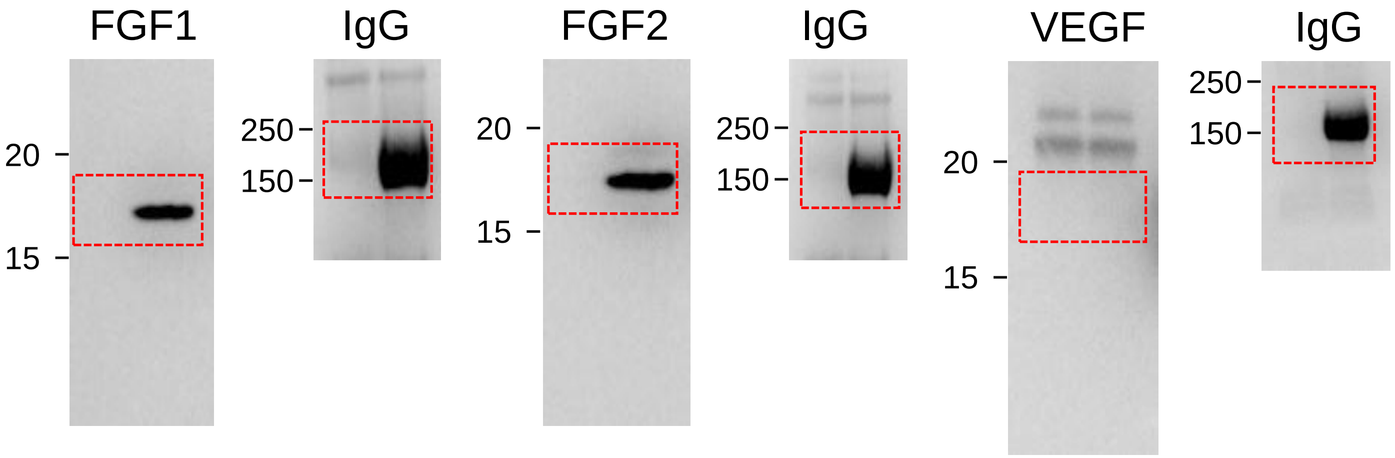

Supplementary Fig.5c

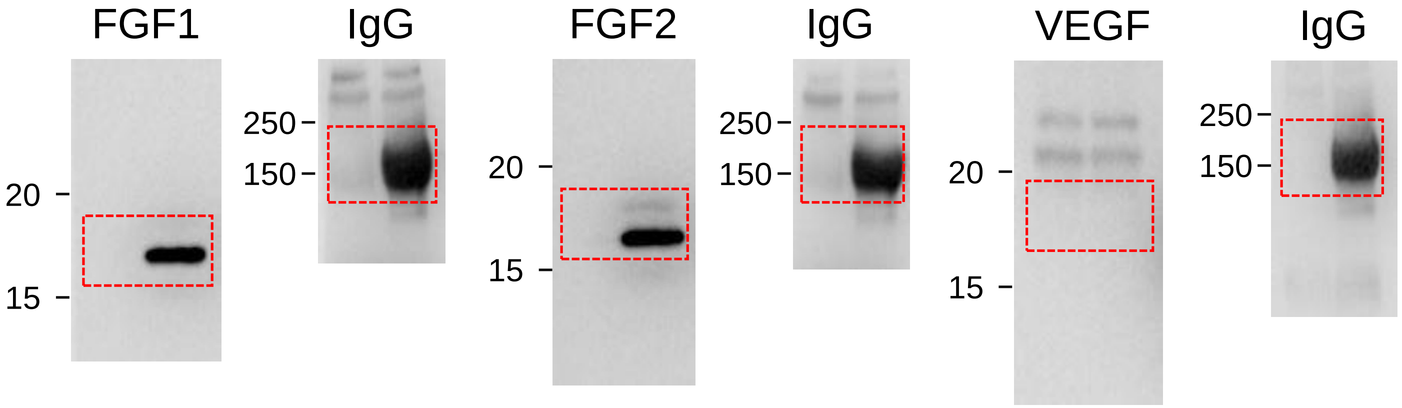

Supplementary Fig.8 (continued)

Supplementary Fig.6e

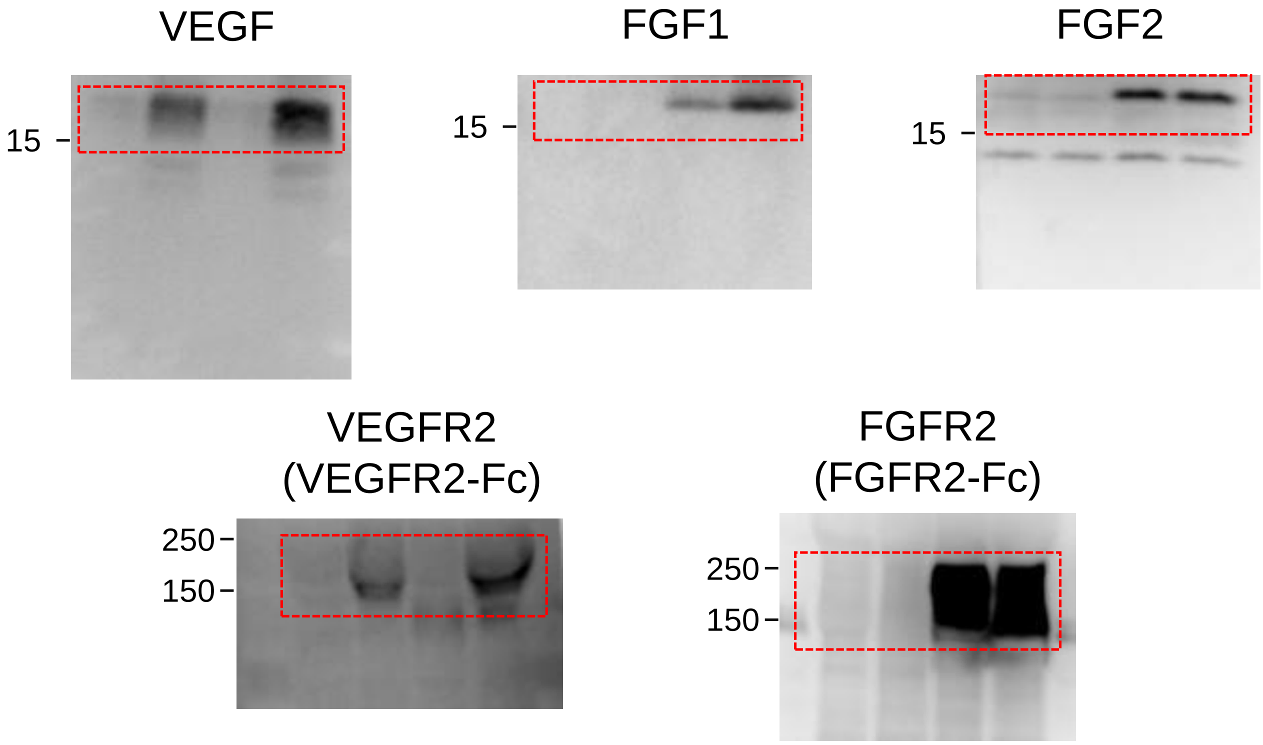

Supplementary Fig.6f

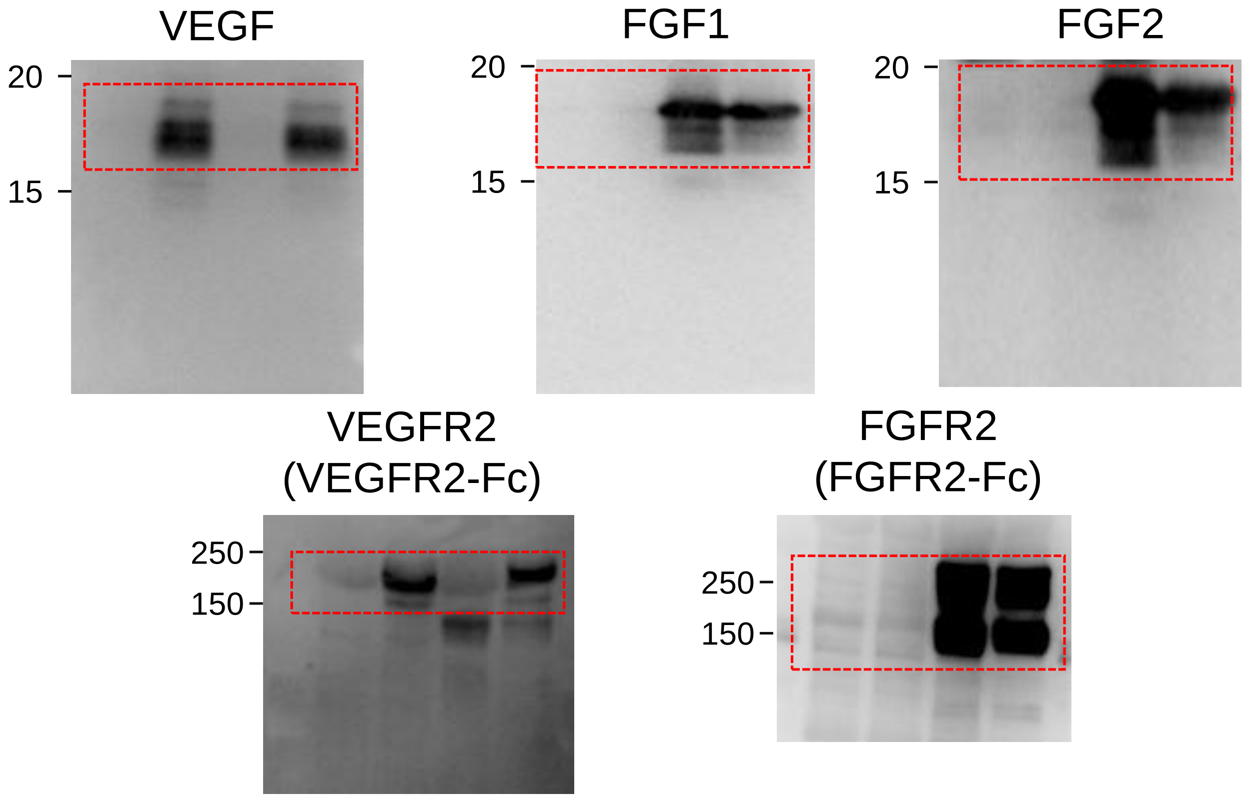

Supplement: Supplementary file 1 — Supplementary information. [file 41598_2020_59853_MOESM1_ESM.pdf]
